# DTX3L Inhibits the EMT, Metastasis, and Stem‐Like Features of Gastric Cancer Through Promoting GSK‐3β Dependent SNAI1 Decay

**DOI:** 10.1002/advs.202524036

**Published:** 2026-04-13

**Authors:** Yang Chen, Zhen Li, Jiajia Shen, Jingyu Lin, Xiaoli Zhao, Rui Zhang, Ying Han, Zhen Wang

**Affiliations:** ^1^ Department of Biochemistry Institute of Medicinal Biotechnology Chinese Academy of Medical Sciences & Peking Union Medical College Beijing China

**Keywords:** DTX3L, EMT, gastric cancer, SNAI1, ubiquitination

## Abstract

Gastric cancer remains a leading cause of cancer mortality worldwide, largely due to its high metastatic potential driven by epithelial‐mesenchymal transition (EMT). Here, we identify Deltex E3 ubiquitin ligase 3L (DTX3L) as a previously unrecognized tumor suppressor in gastric cancer. DTX3L expression is markedly reduced in metastatic and mesenchymal‐type gastric cancers and positively correlates with favorable patient prognosis. Functional analyses in cell lines, organoids and animal models demonstrate that DTX3L depletion promotes gastric cancer cell migration, invasion, stem‐like properties and metastasis, whereas its overexpression exhibits opposite effects. Mechanistically, DTX3L acts as an E3 ubiquitin ligase that directly interacts with and ubiquitinates SNAI1, a master EMT regulator, leading to its GSK‐3β dependent proteasomal degradation. Loss of DTX3L stabilizes SNAI1 and enhances EMT and stem‐like phenotypes. Moreover, we uncover that TGF‐β1‐induced *miR‐135b‐5p* downregulates DTX3L, forming a regulatory axis that promotes EMT. Collectively, our findings reveal a novel DTX3L‐SNAI1 signaling pathway governing EMT and metastasis in gastric cancer, providing mechanistic insight and suggesting DTX3L as a potential prognostic biomarker and therapeutic target.

## Graphical abstract

This research identifies DTX3L as a critical tumor suppressor that inhibits epithelial‐mesenchymal transition (EMT), invasion and metastasis in gastric cancer. By functioning as an E3 ubiquitin ligase, DTX3L targets the master EMT regulator SNAI1 for GSK‐3β‐dependent proteasomal degradation. These findings uncover a novel TGF‐β1/*miR‐135b‐5p*/DTX3L signaling axis, offering a promising therapeutic target to combat cancer stem‐like features and progression.

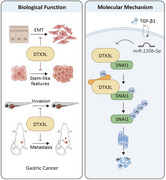

## 1 Introduction

Gastric cancer remains one of leading causes of cancer incidence and mortality worldwide [1]. Most patients are diagnosed at advanced stages, resulting in poor prognosis primarily due to rapid progression and metastasis. A central driver of metastasis is epithelial‐mesenchymal transition (EMT), a reversible process in which epithelial cells lose polarity and adhesion while acquiring mesenchymal traits and motility [2, 3]. Hallmarks include loss of epithelial markers such as E‐Cadherin and gain of mesenchymal markers including N‐Cadherin and vimentin [2, 3]. Loss or mutation of *CDH1*, the gene encoding E‐Cadherin, promotes hereditary diffuse gastric cancer, highlighting the pivotal role of EMT in disease progression [4].

Protein homeostasis is tightly regulated by post‐translational modifications, particularly ubiquitination mediated by the ubiquitin‐proteasome system (UPS) [5]. Despite the recognized importance of EMT in gastric cancer metastasis, the mechanisms governing EMT‐related protein homeostasis remain poorly defined.

In recent years, the Deltex (DTX) protein family has gained increasing attention for its emerging roles in cancer [6]. DTX3L, initially identified in diffuse large B‐cell lymphoma as a binding partner of PARP9, is a member of the DTX family and possesses intrinsic RING‐type E3 ubiquitin ligase activity [7]. DTX3L mediates substrate‐specific ubiquitination to regulate DNA damage responses, interferon signaling, endosome sorting, and protein turnover, and is itself subject to UPS‐mediated degradation [7, 8, 9, 10, 11]. Whether DTX3L targets additional substrates and how this impacts tumor biology remains largely unexplored.

Emerging evidence suggests that DTX3L is upregulated in various cancer types, including lymphoma, glioma, melanoma, and cervical cancer cells [9, 12, 13, 14], where it promotes survival and chemoresistance through pathways such as FAK‐PI3K‐AKT [15], and suppresses IRF1 signaling via interactions with PARP9 and PARP14 to enhance prostate cancer proliferation [16]. In triple‐negative breast cancer, DTX3L stabilizes endothelial lipase (LIPG) to maintain the DTX3L‐LIPG‐ISG15 axis, promoting cell survival and migration, and serves as a negative feedback regulator in all‐trans retinoic acid‐mediated growth inhibition [17, 18]. However, whether DTX3L contributes to gastric cancer progression and metastasis, and whether additional substrate modulates its function in tumors, has not been investigated, highlighting a critical knowledge gap that warrants further study.

In the present work, we report that DTX3L inhibits the EMT, invasion, metastasis, as well as stem‐like features of gastric cancer. Mechanistically, DTX3L functions as an unidentified E3 ubiquitin ligase that specifically interacts with SNAI1, a critical regulator of EMT, and targets the substrate for GSK‐3β dependent decay via UPS.

## 2 Results

### 2.1 DTX3L Expression is Negatively Correlated with EMT Traits in Gastric Cancer, with Its Depletion Enhancing Metastasis in Zebrafish Model

**FIGURE 1:**
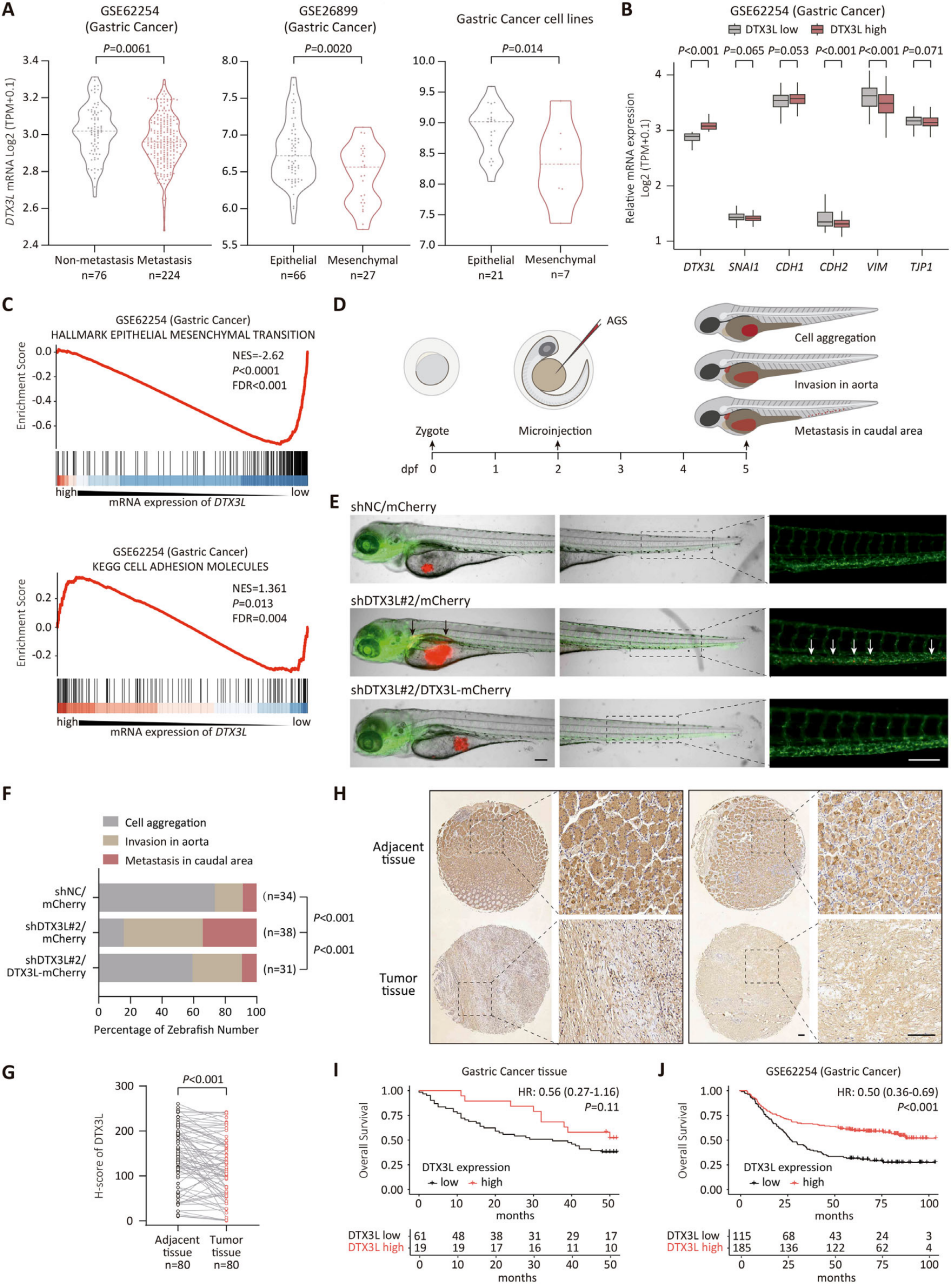
DTX3L expression is negatively correlated with EMT traits in gastric cancer, with its depletion enhancing metastasis in zebrafish tumor model. (A) *DTX3L* mRNA expression levels in GSE62254, GSE26899, and a publicly available dataset of Gastric Cancer cell lines in CCLE. (B) Expression levels of *DTX3L* and key epithelial mesenchymal transition (EMT) markers in the gastric cancer data from GSE62254 stratified by the *DTX3L* expression with median cut‐off value. (C) GSEA analysis of GSE62254. NES, normalized enrichment score. FDR, false discovery rate. (D) Schematic diagram illustrating the workflow for the *Tg (fli:EGFP)* zebrafish xenograft model using mCherry‐labeled AGS cells. (E‐F) Observation (E, Scale bar: 200 µm) and quantification (F) of the invasion and metastasis of the indicated groups of AGS cells by fluorescence microscopy, with arrows indicating the invasion and metastasis to aorta and caudal area of zebrafish larva at 5 dpf. The left two panels in (E) show sequentially captured picture of the same zebrafish. (G‐I) Tissue microarray analysis of DTX3L by IHC staining. Quantification (G) and representative images (H, Scale bar: 100 µm) are shown. The Kaplan–Meier plot in (I) demonstrates overall survival of the patients stratified by DTX3L expression with optimal cut‐off of the H‐scores. (J) The Kaplan‐Meier plot showing the overall survival of the patients based on the DTX3L expression in GSE62254. HR, Hazard Ratio.

By analyzing and comparing several different datasets of gastric cancer patients containing metastasis or EMT information in the Gene Expression Omnibus (GEO) and The Cancer Genome Atlas (TCGA), we discovered that the level of *DTX3L* gene is much lower in metastatic or mesenchymal gastric cancer cohorts compared to the non‐metastatic or epithelial counterparts (Figure 1A; Figure S1A). Analysis of multiple gastric cancer cell lines in the Cancer Cell Line Encyclopedia (CCLE) cell database also reveals a great reduction of *DTX3L* expression in mesenchymal‐type cells relative to the epithelial‐type ones (Figure 1A). We extended analysis of *DTX3L* mRNA levels in the datasets of pan‐cancer cohorts and cell lines from TCGA and CCLE, respectively, and observed similar trends as in that in gastric cancer (Figure S1B,C). Moreover, by stratifying the *DTX3L* expression as high and low groups with median cut‐off value in the gastric cancer data from GEO and TCGA, we found that *DTX3L* expression levels in general positively correlate with that of epithelial marker genes (*CDH1* and/or *TJP1*), while negatively correlates with mesenchymal genes (*CDH2* and/or *VIM*) (Figure 1B; Figure S1D) [19]. Supportively, gene set enrichment analysis (GSEA) from the GEO and TCGA gastric cancer cohorts showed that the expression levels of *DTX3L* negatively and positively correlate with the enrichment levels of EMT pathway and cell adhesion pathway, respectively (Figure 1C; Figure S1E). Thus, these bioinformatic data suggest a possibly negative regulation for DTX3L toward the EMT and metastasis of gastric cancer.

We next investigated whether DTX3L may affect the metastasis of gastric cancer in the *Tg (fli:EGFP*) zebrafish model by establishing mCherry‐labeled AGS cells stably expressing shRNA negative control (shNC), shDTX3L, or shDTX3L with rescued DTX3L (Figure S1F). The knockdown efficiencies of two different shRNAs for DTX3L were validated by immunoblotting (IB) analysis (Figure S2A), and we used shDTX3L#2 for zebrafish experiments. Cells were injected into the yolk sac of 2 day‐post‐fertilization (dpf) zebrafish embryos to establish xenografts, and fluorescence imaging of the zebrafish larva was captured at 5 dpf (Figure 1D). DTX3L‐depleted cells were more easily disseminated into zebrafish circulation, with more metastasis observed in the caudal area of zebrafishes, when compared with the control group; upon re‐expression of DTX3L in the shDTX3L group cells, the invasion and metastasis of gastric cancer were markedly reduced (Figure 1E,F). Moreover, the cancer cells lacking DTX3L exhibited higher fluorescence intensity relative to the control group, while DTX3L re‐expression markedly suppressed the trend (Figure S1G). These results support both anti‐metastatic and anti‐proliferative effects of DTX3L in gastric cancer.

We further analyzed DTX3L protein levels in a tissue microarray containing eighty gastric cancer samples and adjacent counterparts by immunohistochemistry (IHC), and found a markedly lower trend of DTX3L protein abundance in the tumor tissues relative to the adjacent ones, as illustrated in Figure 1G,H. Moreover, patients with higher DTX3L protein levels from the gastric cancer tissue microarray exhibited a tendency of extended overall survival as compared to that with lower DTX3L levels by Kaplan‐Meier analysis (Figure 1I). Supportively, patients with elevated *DTX3L* gene expression levels demonstrated significantly longer overall survival in gastric cancer cohorts in GEO and TCGA datasets (Figure 1J; Figure S1H). Collectively, these data have demonstrated that DTX3L may function as a biomarker and suppressor of EMT, metastasis, and proliferation for gastric cancer.

### 2.2 DTX3L Inhibits the Migration, Invasion, Proliferation, and Stem‐Like Phenotypes of Gastric Cancer

**FIGURE 2:**
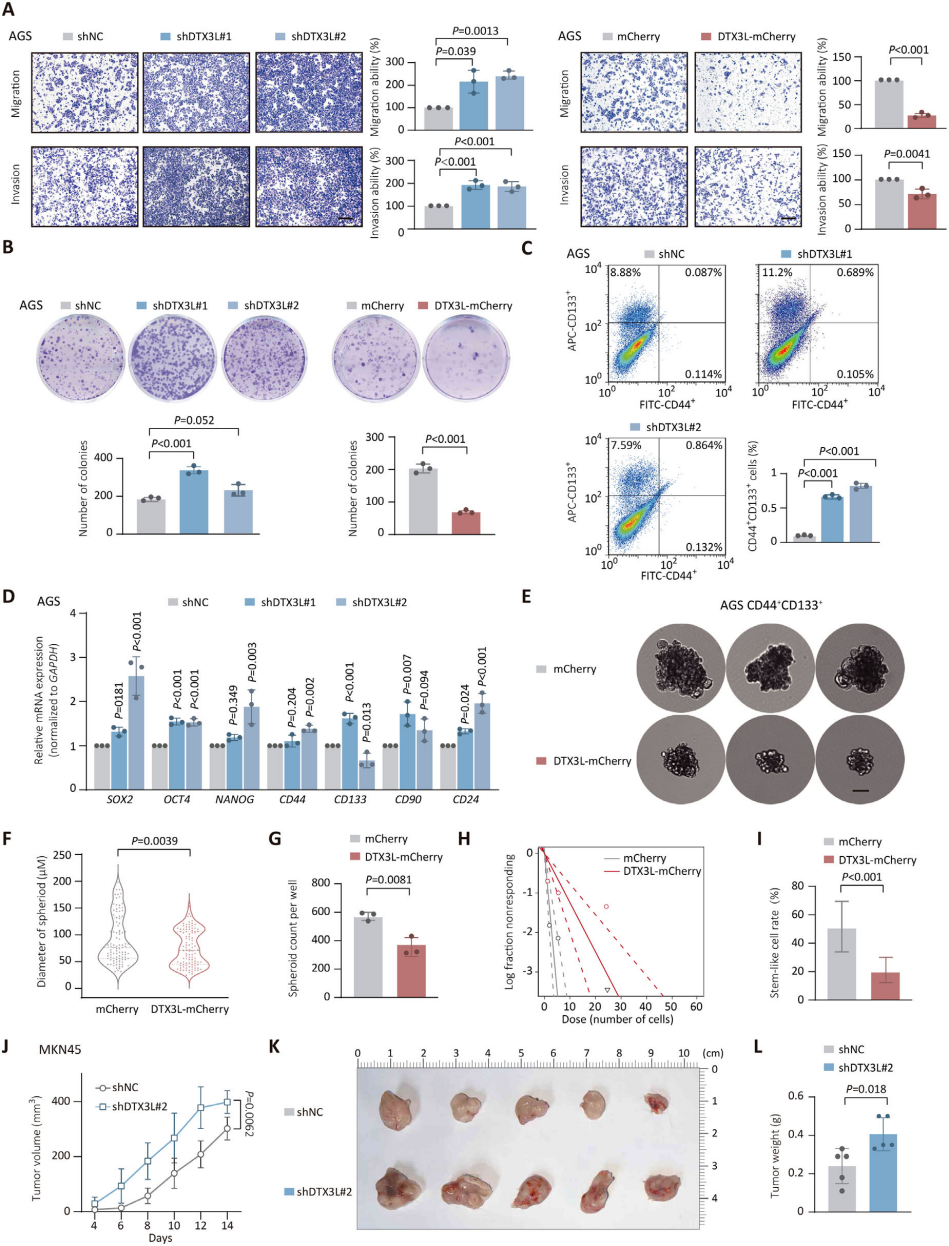
DTX3L inhibits the migration, invasion, proliferation, and stem‐like features of gastric cancer. (A) Migration and invasion assays for AGS cells stably expressing shDTX3L#1, #2, or DTX3L‐mCherry with respective control groups. Scale bar: 200 µm. (B) Colony formation assays of AGS cells as in (A). (C) The proportion of CD44^+^CD133^+^ cells in AGS stably expressing indicated shRNA analyzed by flow cytometry. (D) RT‐qPCR results of stem‐like features associated transcription factors and marker genes in AGS expressing shDTX3L or shNC. P‐values shown represent statistical comparisons relative to the shNC (negative control) group. (E‐I) Stem‐like cell properties of the CD44^+^CD133^+^ AGS cells expressing DTX3L‐mCherry or mCherry. Representative spheroid images (E, Scale bar: 50 µm), quantification of the spheroids size (F) and numbers (G), along with the stem‐like cell frequency (H) and rate (I), are demonstrated. (J‐L) NSG mice were inoculated with MKN45 cells stably expressing either shDTX3L#2 or shNC, and the tumor volume curve was plotted (J), with images (K) and weights of the tumors on the final day shown (L, n = 5).

We next investigated the biological function of DTX3L using human gastric cancer cell lines AGS, HGC27, and MKN45. Transwell assays revealed that DTX3L stably knockdown in AGS and HGC27 cells with either shRNA#1 or shRNA#2 significantly increased the migratory and invasive capacities, while its overexpression exhibited opposite phenotypes (Figure 2A; Figure S2A,B). We also examined the effect of DTX3L on the clonogenic capacity of the gastric cancer cells and found that DTX3L depletion resulted in much enhanced proliferation in AGS and HGC27 cells, whereas its upregulation exhibited opposite effects (Figure 2B; Figure S2C).

As EMT is a biomarker of cancer stem cells [20], we also evaluated whether DTX3L may regulate cancer stem‐like properties. By quantifying the CD44^+^CD133^+^ subpopulation from AGS cells with flow cytometry, a much higher fraction of the CD44^+^CD133^+^ cells were observed in the DTX3L‐depleted cells relative to the control group (Figure 2C). Consistently, RT‐qPCR analysis of stem‐like features related transcription factors (*SOX2*, *OCT4*, *NANOG*) and surface markers (*CD44*, *CD133*, *CD90*, *CD24*) demonstrated a significantly upregulation of most of the genes upon DTX3L knockdown in AGS cells by shRNA#1 or shRNA#2 (Figure 2D). Meanwhile, by isolating and culturing the CD44^+^CD133^+^ subpopulation from AGS cells (Figure S2D), DTX3L overexpression resulted in a significant reduction of both sphere diameter and number of the cells (Figure 2E–G; Figure S2E). Consistently, a pronounced reduction of stem‐like cell frequency was found upon DTX3L overexpression (Figure 2H,I). Interestingly, a down‐regulated trend of *DTX3L* gene expression was observed in the CD44^+^CD133^+^ AGS cells relative to the CD44^−^CD133^−^ counterparts, while *SNAI1*, a known positive regulator of stemness, was found upregulated (Figure S2F) [21]. Thus, these data support that DTX3L is a negative regulator of gastric cancer stem‐like features. Following these findings, we also evaluated the potential function of DTX3L in NSG (NOD*‐scid IL2rγnull*) mouse xenograft model. Expectedly, DTX3L knockdown in MKN45 cell line significantly accelerated the tumor growth in vivo, without affecting the general body weight of the mice (Figure 2J–L; Figure S2G,H). Taken together, these results establish DTX3L as a tumor suppressor that inhibits the migration, invasion, and stem‐like features of gastric cancer.

### 2.3 DTX3L Inhibits EMT and Negatively Regulates SNAI1 Protein Levels via the UPS

**FIGURE 3:**
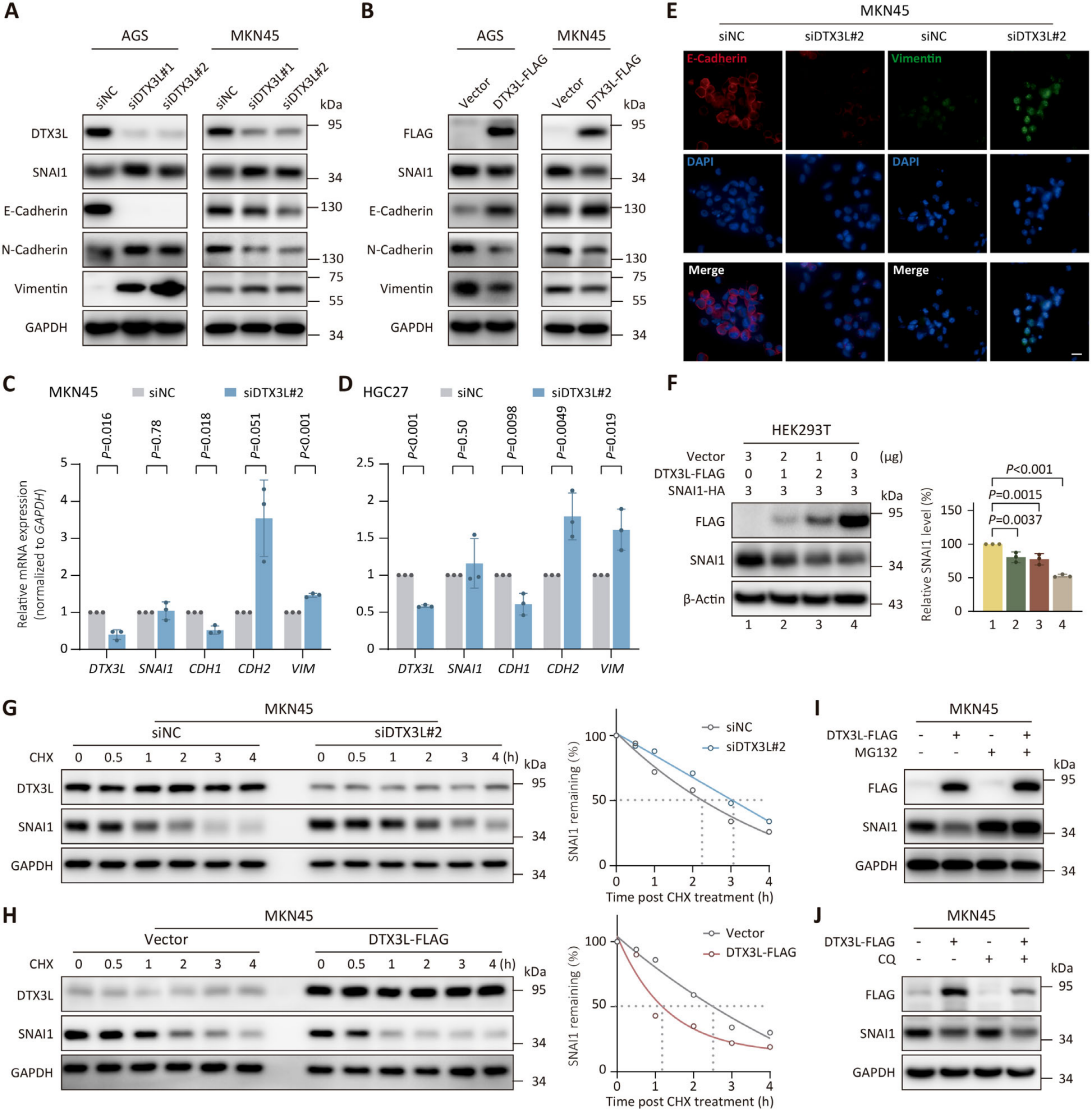
DTX3L inhibits EMT and negatively regulates SNAI1 protein levels via the UPS. (A‐B) IB analyses of the EMT markers in AGS and MKN45 cells upon DTX3L knockdown or overexpression for 48 h. (C‐D) RT‐qPCR analyses of the EMT markers in MKN45 and HGC27 cells upon DTX3L knockdown. (E) IF analysis of E‐Cadherin (red) and Vimentin (green) in MKN45 cells upon DTX3L knockdown for 96 h. Scale bar: 10 µm. (F) HEK293T cells were transfected with increasing doses of DTX3L‐FLAG and 3 µg SNAI1‐HA plasmids for 48 h, followed by IB analyses. The SNAI1 protein levels are shown in the right panel. (G‐H) MKN45 cells transfected with siDTX3L#2 or siNC (G) or DTX3L‐FLAG or Vector (H) for 48 h followed by treatment with 50 µg/ml CHX for indicated time interval before subjected to IB analyses. SNAI1 protein half‐lives are shown in the right panel. (I‐J) MKN45 cells expressing DTX3L‐FLAG or vector for 48 h by transient transfection were treated with 20 µM MG132 (I) or 50 µm CQ (J) for 6 h prior to IB analyses.

Next, we hypothesized that DTX3L negatively regulates EMT. Indeed, DTX3L depletion by siRNAs in AGS and MKN45 cells resulted in increased protein levels of SNAI1, Vimentin, and N‐Cadherin, along with decreased levels of E‐Cadherin, which are positive and negative EMT markers, respectively, whereas DTX3L overexpression exhibited opposite effects (Figure 3A,B). Consistent trends for genes expression of these EMT markers except for *SNAI1* were observed under either DTX3L depleted or overexpressed conditions in AGS, MKN45, and HGC27 cells by RT‐qPCR analysis (Figure 3C,D; Figure S3A,B). By immunofluorescence (IF) staining, E‐Cadherin and Vimentin protein levels were consistently found to be downregulated and upregulated, respectively, upon DTX3L knockdown in MKN45 cells (Figure 3E).

Considering that DTX3L exhibited no effect on *SNAI1* gene expression and that a similarly independent trend for *SNAI1* expression was observed in the gastric cancer data from GEO and TCGA (Figure 1B; Figure S1D), we speculated DTX3L regulates SNAI1 at a post‐transcriptional level. By co‐transfecting HEK293T cells with increasing amounts of DTX3L‐FLAG and SNAI1‐HA expressing plasmids, we found that DTX3L dose‐dependently reduced SNAI1 protein levels (Figure 3F). Consistently, DTX3L depletion markedly extended the protein half‐life of SNAI1, whereas ectopic expression of DTX3L accelerated its turnover in MKN45 and AGS cells (Figure 3G,H; Figure S3C), suggesting that DTX3L promotes SNAI1 decay.

Next, the three gastric cancer cell lines and HEK293T cells were transfected with DTX3L‐FLAG or control vector for 48 h, followed by treatment with either the known proteasome inhibitor MG132 or lysosomal inhibitor chloroquine (CQ) for 6 h. MG132 instead of CQ treatment completely rescued the DTX3L‐mediated SNAI1 reduction (Figure 3I,J; Figure S3D,E), supporting that DTX3L promotes SNAI1 degradation via the UPS. Altogether, these findings prove that DTX3L promotes the proteasomal degradation of SNAI1 and suppresses the EMT process.

### 2.4 DTX3L Interacts with, and Ubiquitinates SNAI1

**FIGURE 4:**
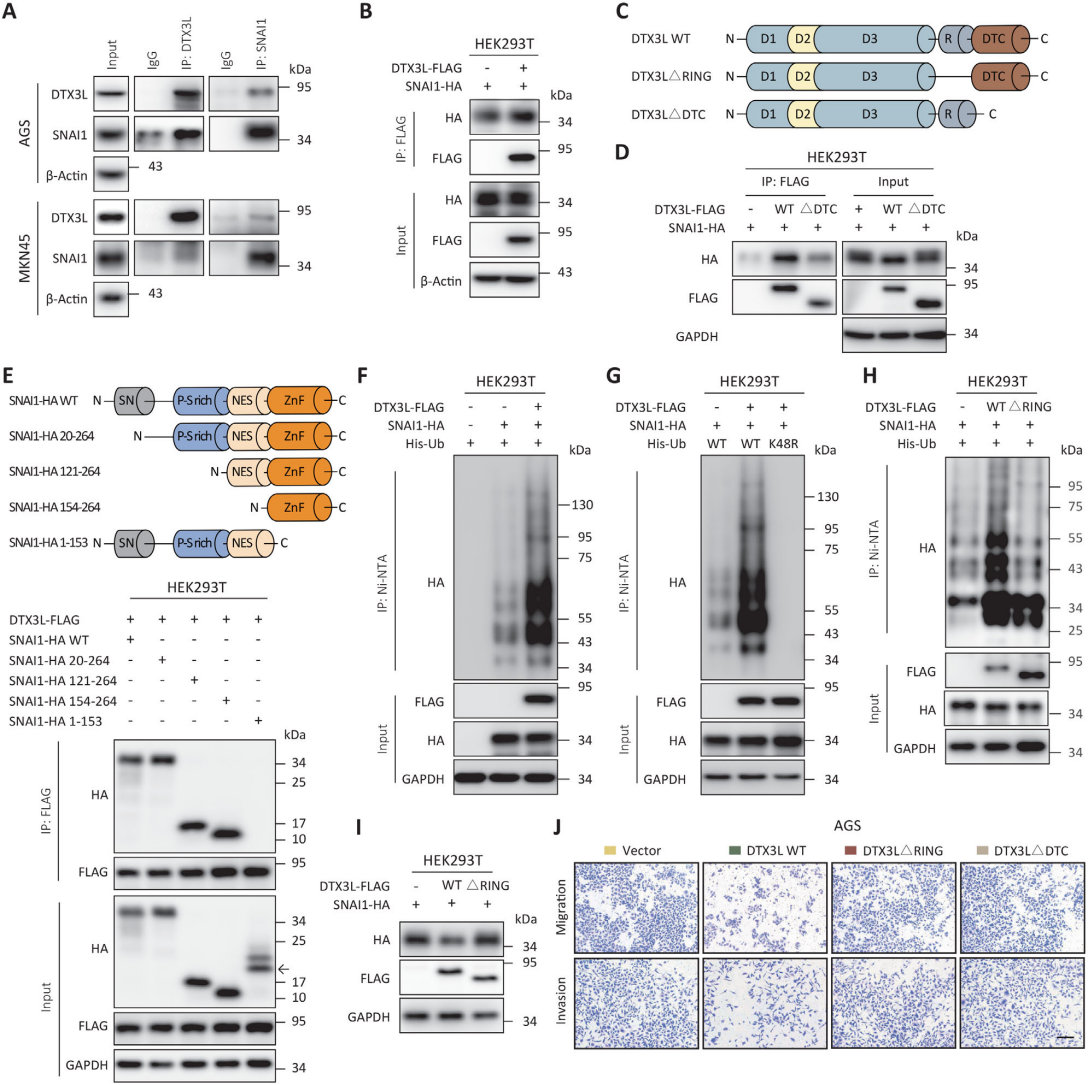
DTX3L interacts with and ubiquitylates SNAI1. (A) AGS and MKN45 cells were subjected to IP using anti‐DTX3L and anti‐SNAI1 antibody or IgG control, followed by IB analyses. (B) IP and IB analyses in HEK293T cells co‐transfected with DTX3L‐FLAG and SNAI1‐HA. (C) Schematic representation of DTX3L deletion mutants. (D) IP and IB analyses in HEK293T cells co‐transfected with indicated plasmids. (E) Upper panel: Schematic representation of SNAI1 deletion mutants. Lower panel: IP and IB analyses of HEK293T cells expressing the SNAI1 mutants. Arrow indicates target band. (F‐H) HEK293T cells were co‐transfected with indicated plasmids. The cells lysates were purified using Ni‐NTA beads and analyzed by IB analyses. (I) IB analyses of HEK293T transfected with indicated plasmids. (J) AGS cells were transfected with wild‐type (WT) or deletion mutants of DTX3L for 24 h, followed by migration and invasion assays. Representative images from three indepedent experiments are shown. Scale bar: 200 µm.

We moved forward to assess whether DTX3L directly binds to SNAI1 by immunoprecipitation (IP) analysis. As found in Figure 4A and Figure S4A, DTX3L binds to SNAI1 under the physiological conditions in AGS, MKN45, and HGC27 cells. Additionally, exogenously expressed DTX3L‐FLAG also interacted with exogenous SNAI1‐HA in HEK293T cells (Figure 4B). These data support that SNAI1 is a bona fide binding partner of DTX3L. To further map the region of DTX3L required for SNAI1 binding, two deletion mutants including the fragment spanning amino acids 1–555 and 556–740 of DTX3L were generated (Figure S4B). Co‐IP results showed that the C‐terminal fragment was indispensable for the DTX3L‐SNAI1 interaction (Figure S4C). Considering the fragment contains the Deltex C‐terminus (DTC) domain that was reported to bind to substrates [22], we further generated a DTC‐domain deletion mutant (DTX3LΔDTC) and found that the domain is indeed critical for the substrate binding (Figure 4C,D). We also defined the functional domain(s) on SNAI1 protein responsible for its interaction with DTX3L by generating serial deletion mutants containing different SNAI1 domains (Figure 4E, upper panel). Pull‐down results demonstrated a critical role of the C‐terminal zinc‐finger domain on SNAI1 to mediate the DTX3L‐SNAI1 interaction (Figure 4E, lower panel). Thus, the two proteins interact with each other via their respective C‐terminus.

To further investigate the underlying mechanism by which DTX3L regulates SNAI1 stability, we co‐expressed DTX3L‐FLAG, SNAI1‐HA, and His‐Ub in HEK293T cells to perform in vivo ubiquitination assay. Expectedly, ectopic expression of DTX3L greatly increased the polyubiquitination of SNAI1 (Figure 4F). We also found ectopic expression of DTX3L facilitated the Lys 48(K48)‐linked polyubiquitin chains on SNAI1 protein, supporting the proteasome‐mediated substrate degradation (Figure 4G) [23]. Consistently, with the interacting regions, the C‐terminus but not N‐terminus deletion mutant of DTX3L failed to enhance the polyubiquitination of SNAI1 (Figure S4D).

As DTX3L is a RING‐type E3 ligase, we then asked whether the RING domain, which is capable of binding to E2 ligases [24], is critical in mediating the ubiquitination activity of DTX3L. Indeed, the RING domain deletion totally abolished the ubiquitination state of SNAI1 compared with the wild‐type (WT) DTX3L (Figure 4H), thus exhibiting negligible effect toward SNAI1 protein levels (Figure 4I). Expectedly, the DTX3LΔDTC mutant defective in substrate binding demonstrated similar results as that of DTX3LΔRING (Figure S4E,F). These data indicate that the C‐terminal DTC and RING domains in DTX3L protein are both critical for its binding and E3 ubiquitin ligase activity toward SNAI1. Supportively, the C‐terminus zinc‐finger deletion mutant of SNAI1 failed to be ubiquitinated and degraded (Figure S4G,H).

We further delved into the potential function of the DTX3L deletion mutants via transwell assays. Unlike the full‐length DTX3L, overexpression of the ΔDTC or ΔRING mutant expectedly failed to suppress the migration and invasion in AGS cells (Figure 4J; Figure S4I). Taken together, our findings support DTX3L as an E3 ubiquitin ligase that binds and ubiquitinates SNAI1 to promote the substrate degradation.

### 2.5 GSK‐3β Dependent Phosphorylation is Essential for DTX3L‐Mediated SNAI1 Degradation

**FIGURE 5:**
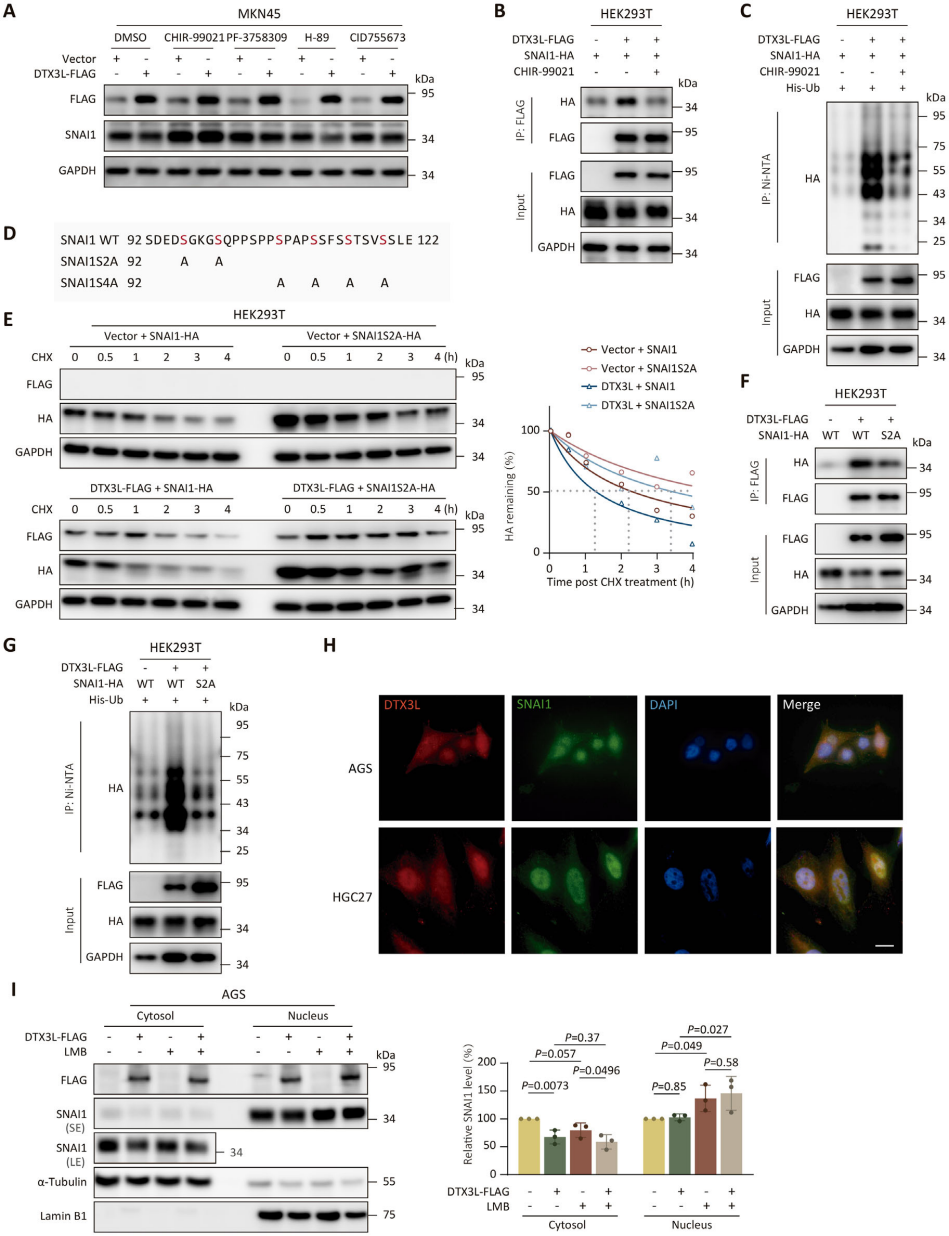
GSK‐3β dependent phosphorylation is essential for DTX3L‐mediated SNAI1 degradation. (A) MKN45 cells were transfected with indicated plasmids for 24 h, followed by another 24 h treatment with respective inhibitors before IB analysis. (B‐C) HEK293T cells were transfected with the indicated plasmids for 24 h and then exposed to 4 µm CHIR‐99021 for additional 24 h before pull‐down with anti‐FLAG IP or Ni‐NTA. (D) Conserved SNAI1 consensus motifs for GSK‐3β phosphorylation, with mutations marked in red. (E) HEK293T cells transfected with indicated plasmids for 48 h followed by treatment with 50 µg/ml CHX for indicated time interval before IB analyses. SNAI1 protein half‐lives are shown in the right panel. (F‐G) Pull‐down with anti‐FLAG IP or Ni‐NTA in HEK293T cells expressing the indicated plasmids. (H) IF analysis of DTX3L (red) and SNAI1 (green) in AGS or HGC27 cells. Scale bar: 10 µm. (I) AGS cells were transfected with DTX3L‐FLAG for 48 h and treated with 100 ng/ml Leptomycin B (LMB) for 2 h. Nucleic and cytoplasmic fractions were isolated and subjected to IB analyses. Protein levels were quantified and plotted in the right panel. SE: short exposure. LE: long exposure.

As SNAI1 has been known to require phosphorylation before recognition and degradation by E3 ubiquitin ligases [25, 26, 27], we next determined whether the phosphorylation is necessary for DTX3L‐mediated SNAI1 degradation. MKN45 cells were treated with several different kinase inhibitors, including CHIR‐99021 (for GSK‐3β), PF‐3758309 (for PAK), CID755673 (for PKD), and H‐89 (for PKA) [25, 26, 27, 28]. As observed in Figure 5A, only the GSK‐3β inhibitor CHIR‐99021 fully restored the DTX3L‐reduced SNAI1 protein levels. Supportively, GSK‐3β inhibition markedly weakened the DTX3L‐SNAI1 interaction as well as DTX3L‐mediated polyubiquitination of the substrate (Figure 5B,C), demonstrating that GSK‐3β dependent phosphorylation is indispensable for DTX3L recognition and ubiquitination of SNAI1.

Previous data have demonstrated that GSK‐3β specifically phosphorylates SNAIL at serine residues in two consensus motifs (as shown in red in Figure 5D) with different function [27]. We generated two SNAI1 mutants including SNAI1S2A (S96A/S100A) and SNAI1S4A (S107A/S111A/S115A/S119A) to examine whether these serine residues are pivotal for the DTX3L‐dependent SNAI1 proteolysis. As seen in Figure 5E and Figure S5A, both mutants exhibited a markedly prolonged half‐life compared with the WT SNAI1 upon DTX3L overexpression in HEK293T cells. However, ectopic expression of SNAI1S2A greatly reduced the DTX3L‐SNAI1 interaction and polyubiquitination of the substrate (Figure 5F,G), whereas that of SNAI1S4A failed to do so (Figure S5B,C). Considering that the GSK‐3β mediated phosphorylation at S96/S100 and S107/S111/S115/S119 is responsible for the substrate cytoplasmic proteolysis and nucleus exportation, respectively [27], the results imply that DTX3L degrades the substrate at the cytoplasmic localization.

Consistent with previous studies [15, 29], we found that DTX3L protein distributed to cytoplasmic and nuclear localization, while SNAI1 predominantly located to the nucleus, by IF staining in AGS and HGC27 cells (Figure 5H). To prove that DTX3L‐mediated SNAI1 degradation occurs at the cytoplasm rather than the nucleus, subcellular contexts of AGS cells overexpressed with DTX3L were separated for IB analysis in the absence and presence of the nucleus export inhibitor leptomycin B (LMB) or MG132. Indeed, DTX3L induced pronounced SNAI1 proteolysis at the cytoplasm rather than the nucleus when comparing the first two lanes from cytoplasmic and nucleic lysates (Figure 5I; Figure S5D), with the effect being greatly rescued by MG132 treatment (Figure S5D). In contrast, treatment with LMB largely trapped more SNAI1 proteins to the nucleus without affecting its cytosolic degradation by DTX3L (Figure 5I). We also found that neither the DTX3L‐SNAI1 interaction nor the DTX3L‐mediated SNAI1 polyubiquitination was affected by LMB (Figure S5E,F). These data confirm DTX3L targets SNAI1 for degradation after the substrate has been exported from the nucleus. Collectively, these findings prove that GSK‐3β dependent phosphorylation is critical for SNAI1 degradation by DTX3L.

### 2.6 SNAI1 Depletion Abrogates the Enhanced Metastasis and Proliferation of DTX3L‐Depleted Gastric Cancer Cells

**FIGURE 6:**
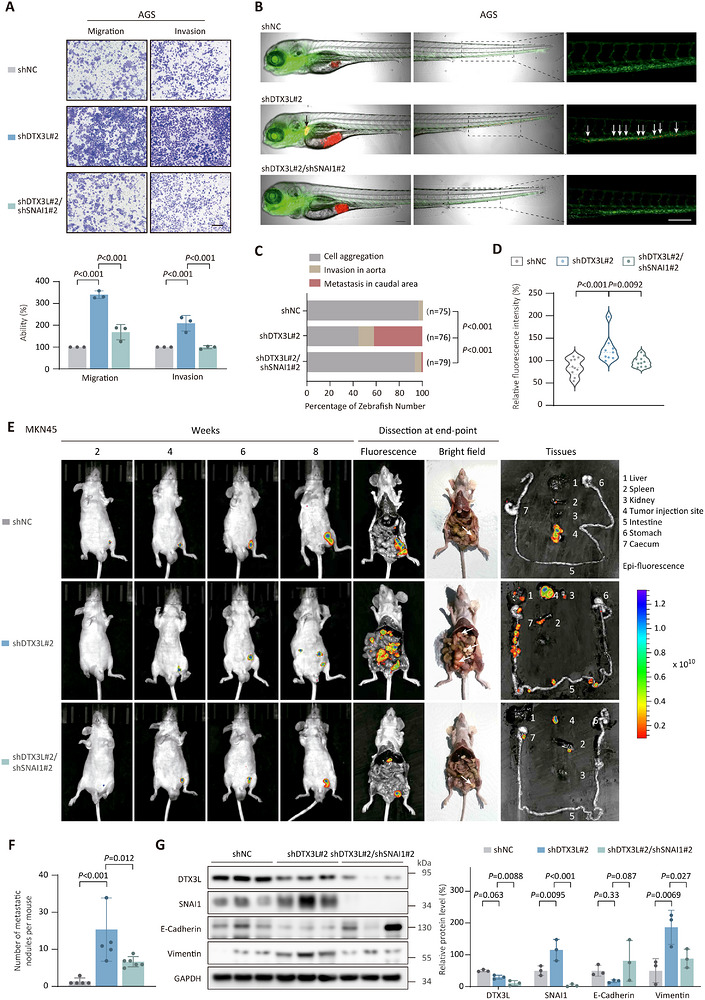
SNAI1 depletion abrogates the enhanced metastasis of DTX3L‐depleted gastric cancer cells. (A) Migration and invasion assays for AGS cells stably expressing indicated shRNA. Representative images of three independent experiments are shown (Scale bar: 200 µm). (B‐D) Observation and quantification of the invasion and metastasis of the indicated groups of AGS cells by fluorescence microscopy in zebrafish xenograft model, with arrows indicating the invasion and metastasis to aorta and caudal area of zebrafish larvae at 5 dpf (B, Scale bar: 200 µm). The numbers of zebrafish with invasion and metastasis were counted (C), and the fluorescence intensity of the aggregated cells were quantified (D, n = 10). The left two panels in (B) show sequentially captured picture of the same zebrafish. (E‐G) Metastatic model of GFP‐labeled MKN45 cells in BALB/c nude mice. The mice were observed regularly during the procedure, with different organs dissected at the end‐point of the 8th week, with arrows indicating the metastatic tumors in abdominal cavity (E). Numbers of metastatic nodules were counted (F, n = 5–6), and the EMT markers levels in metastatic tumors were demonstrated (G).

Next, it is straight forward to determine whether DTX3L exerts its tumor‐suppressive function through degrading SNAI1. After validating the knockdown efficiency of shSNAI1#1 and #2 in DTX3L‐deficient AGS and MKN45 cells using lentiviral‐mediated transduction (Figure S6A,B), we selected shSNAI1#2 for further functional analysis. As shown in Figure 6A, SNAI1 deficiency largely reversed the enhanced migration and invasion caused by DTX3L knockdown in AGS cells by transwell assay. Similarly, using the zebrafish xenograft model as illustrated in Figure 1D, we found simultaneous SNAI1 depletion markedly abolished the increased invasion and metastasis as well as the proliferation of the mCherry‐labeled AGS cells upon DTX3L knockdown (Figure 6B–D). We also performed animal study in mice via intraperitoneal injection of EGFP‐labeled MKN45 expressing shDTX3L#2 in the absence or presence of shSNAI1#2 for eight weeks. Mice from different groups were captured regularly under fluorescence microscopy to observe the cancer metastasis and dissected at the end point of the experiment. Consistently, with the findings observed in zebrafish model, DTX3L knockdown in MKN45 apparently increased the dissemination of the cancer cells and metastatic nodules to different organs in the abdomen of mice, whereas co‐depletion of SNAI1 markedly abrogated the effect (Figure 6E), which was further supported by counting the number of metastatic nodules in the three groups (Figure 6F). The body weights of the three groups of mice remained largely unchanged (Figure S6C). IB analysis of the protein expression of the metastatic tumor tissues in colorectum also proved an increased level of SNAI1 and Vimentin accompanied by decreased level of E‐Cadherin in the DTX3L‐deficient group, which was abrogated by DTX3L and SNAI1 co‐depletion (Figure 6G).

**FIGURE 7:**
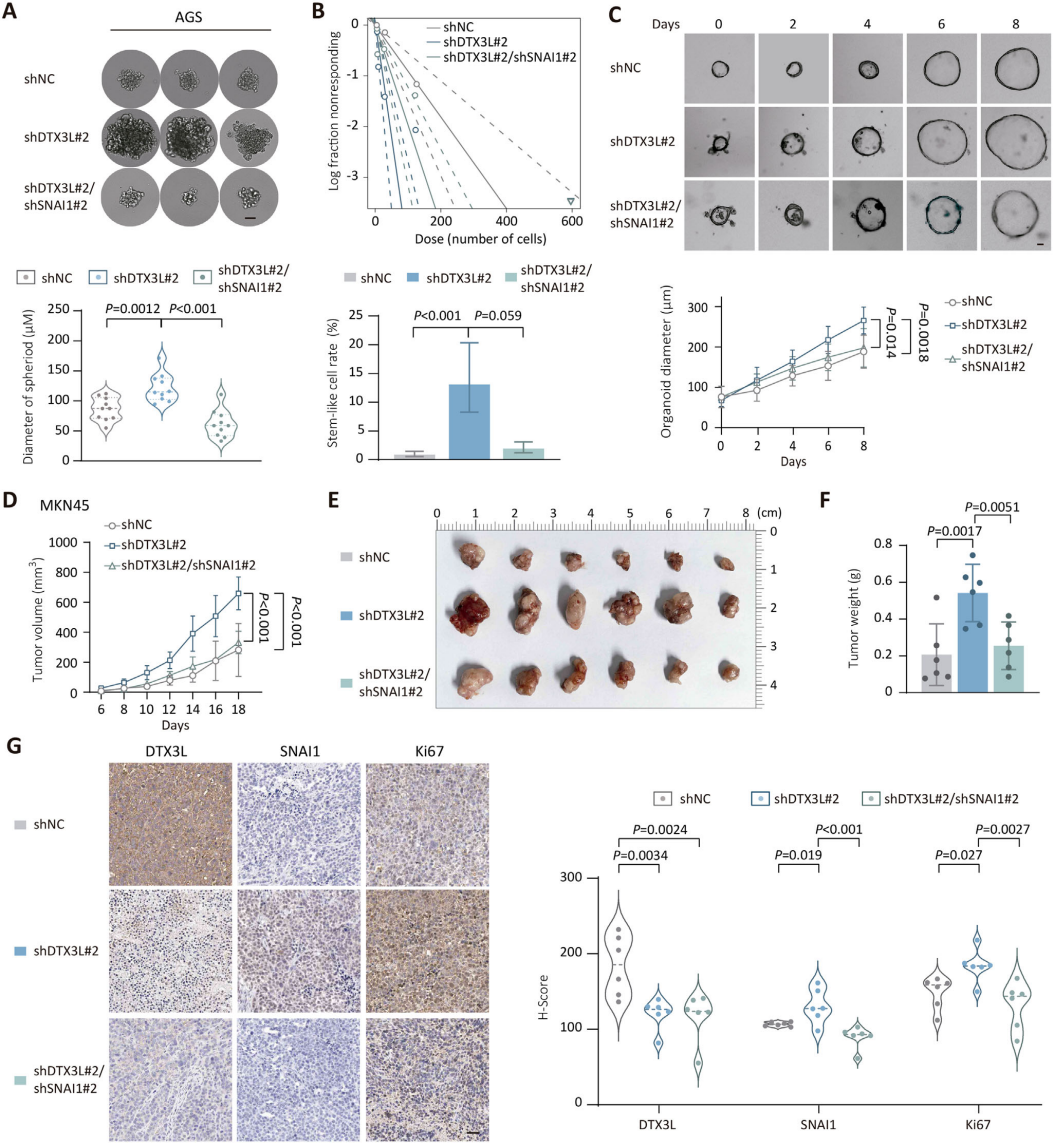
SNAI1 deficiency abolishes the increased proliferation of gastric cancer cells upon DTX3L depletion. (A) Representative spheroid images (Scale bar: 50 µm) and quantification of the spheroids size (n = 10) of AGS cells stably expressing shNC, shDTX3L#2 alone or in combination with shSNAI1#2. (B) Stem‐like cell frequency and rate of the AGS cells as in (A). (C) The proliferation of human gastric cancer organoids. The organoids stably expressing indicated shRNA were photographed at indicated time point (Scale bar: 50 µm), and the sizes of the organoids were quantified (n = 5). (D‐F) BALB/c nude mice were inoculated with indicated groups of MKN45 cells, and the tumor volume curve was plotted (D), with images (E) and weights (F) of the tumors on the final day shown (n = 6). (G) IHC analysis of the indicated proteins expression for tumor tissues from (E). Representative images (Scale bar: 50 µm) and H‐scores are shown for indicated proteins expression.

We then asked whether SNAI1 is also required for DTX3L regulated proliferation and stem‐like phenotypes. Colony formation and growth curve assays revealed that silencing SNAI1 largely abolished the proliferation stimulatory effects conferred by DTX3L depletion in AGS cells (Figure S7A,B). Similarly, the CD44^+^CD133^+^ stem‐like cell fraction was markedly reduced upon co‐depletion of SNAI1 and DTX3L relative to the DTX3L depletion alone (Figure S7C). Sphere‐formation assays further revealed that SNAI1 knockdown greatly attenuated the increase in sphere size induced by DTX3L loss, with a consistent trend of stem‐like cell frequency observed as well (Figure 7A,B).

We also cultured human organoid of gastric cancer and consistently found that DTX3L silencing significantly enhanced organoid proliferation, which was efficiently inhibited upon concomitant SNAI1 knockdown (Figure 7C). Moreover, SNAI1 co‐silencing markedly attenuated the accelerated tumor growth of DTX3L‐deficient MKN45 cells in animal study (Figure 7D–F; Figure S7D). Expectedly, IHC analysis confirmed that both SNAI1 and Ki67 levels were increased upon DTX3L depletion, whereas SNAI1 co‐silencing reversed these effects (Figure 7G). Altogether, these data strongly support that DTX3L functions as a tumor suppressor primarily through promoting SNAI1 proteolysis.

### 2.7 *miR‐135b‐5p*, Induced by TGF‐β1, Suppresses *DTX3L* Expression

**FIGURE 8:**
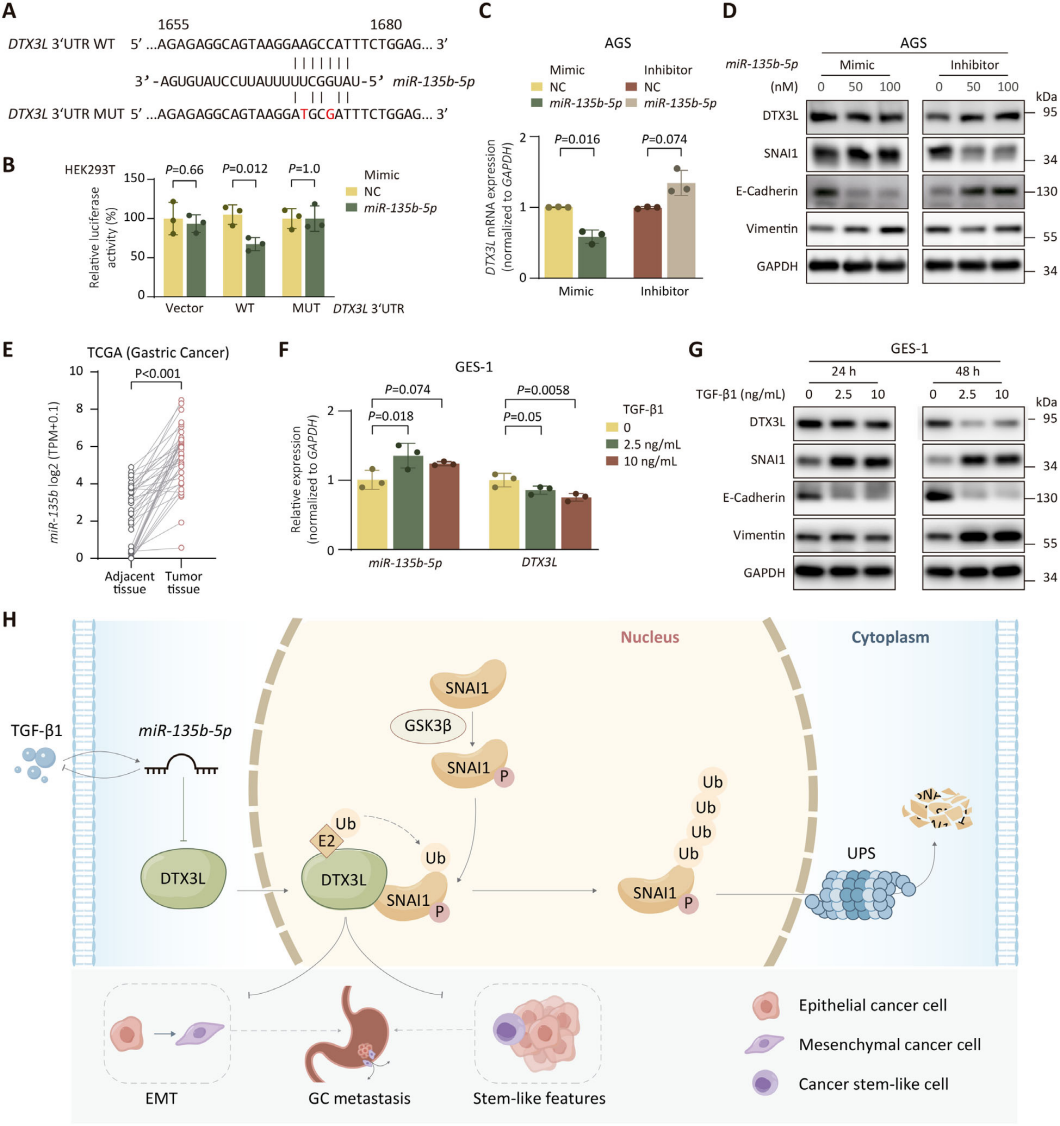
TGF‐β1‐induced *miR‐135b‐5p* suppresses *DTX3L* expression. (A) Putative binding sites of the *miR‐135b‐5p* seed sequence within the 3' UTR of *DTX3L*, with the mutation site labeled in red. (B) Dual‐luciferase reporter assay in HEK293T cells after transfection with 3' UTR of *DTX3L* (WT or MUT) or pmirGLO empty vector and treatment with 50 nM of *miR‐135b‐5p* mimic for 24 h. (C) AGS cells were transfected with 50 nM *miR‐135b‐5p* mimic or inhibitor for 48 h, and *DTX3L* mRNA levels were quantified by RT‐qPCR. (D) IB of the protein levels in AGS cells transfected with *miR‐135b‐5p* mimic or inhibitor for 48 h. (E) *miR‐135b* expression levels in forty‐one paired gastric cancer and adjacent tissues in TCGA. (F) RT‐qPCR analyses of *miR‐135b‐5p* and *DTX3L* expression in GES‐1 cells treated with TGF‐β1 for 48 h. (G) IB analyses of the indicated protein levels in GES‐1 upon TGF‐β1 treatment. (H) The working model that illustrates the role of DTX3L in regulating EMT, proliferation, and stem‐like phenotypes of gastric cancer (GC) by degrading SNAI1 via the ubiquitin‐proteasome system (UPS).

Given that the mRNA levels of *DTX3L* are higher in cancer relative to the normal tissues, including gastric cancer [30], which are inconsistent with the protein levels as found in Figure 1H, we speculated that DTX3L might be regulated at a post‐transcriptional level in cancer. To explore the potential mechanism, we searched TargetScan for potential miRNA binding sites to the *DTX3L* mRNA sequence and identified a predicted *miR‐135b‐5p* binding site within its 3’ UTR region (Figure 8A). We cloned the *DTX3L* 3' UTR containing putative *miR‐135b‐5p* binding sites or a mutant region into a reporter vector pmirGLO (Figure 8A), and performed dual‐luciferase reporter assay to investigate whether *miR‐135b‐5p* directly binds *DTX3L* and regulates its expression. Expectedly, we found *miR‐135b‐5p* treatment significantly inhibited the luciferase activity of WT but not mutant *DTX3L* 3’ UTR reporter vector (Figure 8B), suggesting that *miR‐135b‐5p* specifically binds to the *DTX3L* 3’ UTR. We transfected *miR‐135b‐5p* mimic or inhibitor to AGS and the gastric mucosal epithelial cell line GES‐1 to find out whether the miRNA directly affects DTX3L levels. Indeed, *miR‐135b‐5p* mimic greatly reduced the DTX3L mRNA and protein levels, accompanied by increased SNAI1 and Vimentin as well as decreased E‐Cadherin protein abundances, whereas the inhibitor treatment exhibited opposite effects (Figure 8C,D; Figure S8A). Interestingly, analysis of the gastric cancer dataset in TCGA revealed a significant upregulation of *miR‐135b* in tumors relative to matched adjacent tissues (Figure 8E), possibly explaining why the DTX3L protein levels are lower in tumoral tissues.

Lastly, we delved into determining why the *DTX3L* mRNA levels are lower in mesenchymal‐type than that in epithelial‐type cancer cells as found in Figure 1A and Figure S1A,B. Since TGF‐β1 plays a central role in driving EMT [31], we hypothesize that TGF‐β1 may downregulate *DTX3L* expression during EMT induction. Expectedly, TGF‐β1 treatment in GES‐1 cells dose‐dependently reduced the mRNA and protein levels of DTX3L, accompanied by consistent changes of EMT markers (Figure 8F,G). Notably, TGF‐β1 markedly upregulated *miR‐135b‐5p* expression (Figure 8F). Considering *miR‐135b* was found to suppress TGF‐β1 signaling pathway in gastric cancer [32], we investigated the effect of *miR‐135b‐5p* in TGF‐β1 signaling. As seen in Figure S8B, *miR‐135b‐5p* treatment in AGS cells greatly inhibited the TGF‐β1‐induced Smad2/3 translocation into the nucleus analyzed by IF staining. These data support an unidentified TGF‐β1‐*miR‐135b‐5p* axis regulation exists upstream of DTX3L expression, in which *miR‐135b‐5p* negatively regulates TGF‐β1 signaling pathway (Figure 8H).

## 3 Discussion

DTX3L has been identified as a key E3 ubiquitin ligase that regulates diverse biological processes with different substrates including TBK1, p53, TIRR, and Histone H4 [10, 11, 33, 34]. In the present study, we identified DTX3L as a pivotal regulator of EMT and stem‐like features in gastric cancer and demonstrated its strong association with patient metastasis and prognosis. Functional assays in both cultured cells and animal models of zebrafish and mice proved that DTX3L silencing markedly enhanced the migratory, invasive, proliferative, and stem‐like properties of gastric cancer cells (Figs. 1&2). Mechanistically, DTX3L acts as an E3 ubiquitin ligase targeting SNAI1 for degradation, a process that requires GSK‐3β mediated phosphorylation of SNAI1 to direct it to the UPS (Figure 8H).

Most studies to date have emphasized that DTX3L exerts oncogenic activities in cell proliferation, apoptosis resistance, chemoresistance, and DNA damage repair, thereby facilitating tumor progression, as reviewed [35], with upregulated DTX3L expression seen across multiple malignancies [30]. However, focusing on gastric cancer where DTX3L remains poorly characterized, our findings reveal an unrecognized tumor‐suppressive role for DTX3L in EMT, metastasis, and stem‐like features regulation. Supportively, a previous study demonstrated that the translocation of DTX3L in nucleus reduced the EMT characteristics of Vemurafenib‐resistant melanoma cell line SKMEL28, although the underlying mechanism lacked [36]. Interestingly, additional pieces of indirect evidence also have suggested DTX3L exerts tumor‐suppressive function, which reported a mutually negative regulation between DTX3L and USP28 [37], with the latter protein promoting the proliferation and migration of gastric cancer cells [38]. Moreover, DTX3L depletion was found to increase the levels of both oncogenic and tumor‐suppressive proteins including HIF‐1α, p53, and MYC [37]. Thus, these findings together with our data have demonstrated a complicated and possibly context‐dependent function of DTX3L in cancer.

In the present work, we identified SNAI1 as a novel substrate of DTX3L and demonstrated that DTX3L suppresses EMT by promoting SNAI1 degradation through K48‐linked polyubiquitination (Figs. 3&4). As a master regulator of EMT, cancer metastasis, and stemness, SNAI1 is consistently upregulated in a broad spectrum of human cancers, whose elevated expression normally correlates with poor clinical outcomes, including gastric cancer [39]. Being an intrinsically very unstable protein, SNAI1 is tightly regulated by UPS with multiple E3 ligases being found in cancer, including F‐box proteins such as FBXO11, FBXL5, and FBXW1 (β‐TRCP1) [25, 27, 40]. Here, we establish DTX3L as a previously unrecognized E3 ligase governing SNAI1 proteolysis in gastric cancer, therefore adding the protein to the known E3 ligases family that control the pivotal EMT regulator's protein homeostasis.

Although DTX3L shuttles between the nucleus and cytoplasm [16], its colocalization with SNAI1 occur primarily within the nucleus (Figure 5H). By mutation, protein half‐life, and subcellular fractionation analyses, we further showed that DTX3L‐mediated SNAI1 degradation predominantly takes place in the cytoplasm through a two‐step mechanism: DTX3L binds and ubiquitinates SNAI1 in the nucleus, after which the polyubiquitinated SNAI1 is exported to the cytoplasm for proteasomal degradation (Figure 5; Figure S5). Consistent with the previous study [27], our data demonstrate GSK‐3β dependent phosphorylation at S96/S100 on SNAI1 is crucial for its recognition and ubiquitination by DTX3L, whereas the phosphorylation at S107/S111/S115/S119 are indispensable for nuclear export but not for DTX3L binding (Figure 5; Figure S5). These results indicate DTX3L controls SNAI1 protein homeostasis by a similar regulatory mechanism as FBXW1 does in GSK‐3β dependent way [27]. Whether the two E3 ligases promote SNAI1 degradation in a competitive or cooperative manner remains obscure. Structurally, our data reveal that the DTC domain of DTX3L is essential for its binding and ubiquitination of SNAI1. As the DTC domain represents a conserved C‐terminal feature of all DTX family members [22], the present findings provide new mechanistic insights for potential function of other DTX family E3 ligases in cancer.

A noteworthy finding of the present work is that *DTX3L* gene expression is negatively regulated by *miR‐135b‐5p*, which is upregulated in gastric cancer (Figure 8), possibly explaining the discrepancy between DTX3L protein and mRNA levels in gastric cancer. Supportively, *miR‐135b* reportedly promotes EMT and oncogenesis including gastric cancer [32, 41]. Our experiments further revealed that TGF‐β1 as a pivotal EMT driver induces the *miR‐135b‐5p* expression, therefore forming the upstream regulatory axis of DTX3L. Interestingly, *miR‐135b* has been found to inhibit the TGF‐β1 and Smad activation [32, 42]. As we consistently revealed a negative role of *miR‐135b‐5p* in TGF‐β1 signaling pathway, these data have supported a negative feedback regulation exists in the TGF‐β1‐*miR‐135b‐5p* axis. Thus, despite the established transcriptional regulation in TGF‐β1 mediated EMT, our work demonstrated an alternative pathway of regulation for TGF‐β1 at post‐transcriptional levels.

Collectively, our study uncovers a previously unrecognized layer of protein homeostasis regulation in gastric cancer and provides a conceptual basis for developing DTX3L as a prognostic biomarker and/or therapeutic target for metastatic cancer.

## 4 Experimental Section

### 4.1 Cell Lines

AGS, MKN45, HGC‐27, and HEK293T cell lines were purchased from the National Infrastructure of Cell Line Resources. GES‐1 cell line was purchased from the Enogene Biotechnology Company. AGS, HEK293T, GES‐1 cell lines were cultured in DMEM (SH30022.01, Hyclone) with 10% FBS (#40130ES, Yeasen). MKN45 and HGC27 cells lines were cultured in RPMI 1640 (SH30809.01, Hyclone) with 10% FBS. All cell lines were incubated at 37°C in 5% CO_2_ incubator. All cell lines were authenticated by STR profiling on a regular basis every half year.

### 4.2 Chemicals and Antibodies

MG132 (S2619), Cycloheximide (CHX, S7418), Chloroquine (CQ, S6999), CHIR‐99021 (S1263), PF‐3758309 (S7094), H‐89 (S1582), and CID755673 (S7188) were obtained from Selleck. TGF‐β1 (HY‐P7118), Puromycin (HY‐B1743), and Geneticin (HY‐17561) were from MCE. Leptomycin B (LMB, 50503ES08) and Phalloidin (#40734ES75) were from Yeasen. DAB (3,3′diaminobenzidine) (ZLI‐9018), DAPI (ZLI‐9557) were from ZSGB‐BIO. Polybrene (C0351) were from Beyotime. Detailed information about antibodies used in IB, IHC, IF, IP, and flow cytometry is listed in Table S1.

### 4.3 Vectors and siRNAs

pCMV6‐DTX3L‐FLAG (RC207944) and pCMV6‐Entry Vector (PS100001) were obtained from OriGene. pcDNA3.1‐SNAI1‐HA (#31697) was obtained from Addgene. The mutants of pcDNA3.1‐DTX3L/SNAI1 were obtained from Sangon. The His‐tagged ubiquitin expression plasmids His‐Ubiquitin (His‐Ub) and His‐UbK48R were gifted by Prof. Hu Ronggui at the Hangzhou Institute for Advanced Study, the University of Chinese Academy of Sciences. Lentiviral expression plasmids including pLV3‐shRNA‐EF1 control (P23903), pLV3‐DTX3L‐shRNA#1 and #2 (P53845 and P53851), pLV3‐SNAI1‐shRNA#1 and #2 (P42427 and P41615), pLV2‐mCherry control (P62285), pLV2‐DTX3L‐mCherry (P56747) and pmirGLO (P0198) were obtained from MiaoLing. pLenti‐gRNA‐EGFP (D8301) were obtained from Beyotime. The DTX3L‐targeted siRNAs were synthesized by RIBOBIO. The *miR‐135b‐5p* mimic/inhibitor and negative control were synthesized by GenePharma. The target sequences of siRNAs, shRNAs, and miRNA mimic or inhibitor are listed in Table S2.

### 4.4 Cell Transfection and Infection

For transient transfection, cells were transfected with plasmids and siRNAs using DNA transfection reagent (TF20121201, Neofect) or TransIntro EL Transfection Reagent (FT201‐02, Transgen) for 48 h according to the manufacturer's protocols. For cell transduction, lentivirus vectors were transfected into HEK293T cells with package and envelope plasmids (psPAX2 and pMD2.0G) to produce viruses for stable gene knockdown or expression. At 72 h after transfection, the supernatants containing viruses were collected for transduction, and stable cells were selected.

### 4.5 Tumor Model in Zebrafish

The zebrafish xenograft and metastasis model was conducted following the description provided in the literature [43]. Briefly, 400 AGS cells were micro‐injected into the *Tg (fli:EGFP*) zebrafish embryos at 2 dpf. At 5 dpf, the zebrafish larvae were observed under fluorescent microscope (DMi8, Leica). The fluorescence intensity was quantified with ImageJ.

### 4.6 Tumor Model in Mice

For xenograft model in mice, six‐ to eight‐week‐old male NSG mice (Nuohang Biotechnology) or BALB/c nude mice (SPF Biotechnology) divided into two (n = 5 for each group of NSG mice) or three groups (n = 6 for each group of BALB/c mice) by body weight using a stratified randomization approach in two rounds of independent experiments. 2 × 10^6^ MKN45 cells were subcutaneously inoculated into the right axilla of the mice. The body weight of the mice, length, and width of tumors were measured every two days during the experiment. On the 14th (NSG) or 18th (BALB/c) day post‐implantation, the mice were euthanized, and the tumors were harvested, photographed, and weighed.

Establishment of tumor metastasis model in mice was conducted as stated previously [44]. Briefly, six‐week‐old male BALB/c nude mice were divided into three groups (n = 6 for each group) by body weight using a stratified randomization approach to ensure balanced distribution across experimental groups. 100 µl of cell suspension containing 1 × 10^6^ MKN45 cells was injected into the peritoneal cavity of mice. Tumor progression and metastasis are monitored through bioluminescence imaging system (IVIS SPECTRUM, PerkinElmer) every week, with the body weight measured regularly. At the 8th week post‐injection, the mice were euthanized and dissected for visualization with the bioluminescence imaging system. Metastatic nodules in abdomen were counted, and tumor tissues in colorectum were collected for IB analysis.

All animal experiments were reviewed and approved by the Animal Experimentation Ethics Committee of the Institute of Medicinal Biotechnology (IMB), Chinese Academy of Medical Sciences (CAMS), and were performed in compliance with the regulations and operational procedures of IMB, CAMS. The measurements and analyses were performed in a double‐blinded manner.

### 4.7 Transwell and Proliferation Assays

AGS (5 × 10^4^ cells/well) or HGC27 (3 × 10^4^ cells/well) cells were suspended in serum‐free culture medium and placed into the upper Transwell or Matrigel invasion chamber, with full serum medium placed in the lower chamber. After incubation for 48 h, the chambers were fixed, stained, and quantified as described previously [45]. The colony formation and growth curve assay were performed as described previously [46].

### 4.8 IB, IP, and IF Analysis

IB analysis was conducted as previously described [47]. Nucleic and cytoplasmic fractions were prepared with a commercial kit according to the manufacturer's instructions (P0027, Beyotime). For protein half‐life analysis, cells were treated with 50 µg/ml CHX for the indicated times before IB analysis. After quantification and normalization, decay curves were fitted and half‐lives were calculated in GraphPad Prism. For IB quantification, band intensity was measured and quantified with ImageJ. IP analyses were performed according to established protocols [46]. For IF analysis, cells were seeded on coverslips and fixed with 4% paraformaldehyde. The proteins were stained with respective antibodies, followed by staining with DAPI (for nuclei) or Phalloidin (for F‐actin). Images were captured using fluorescence microscope (Delta Vision, GE Healthcare).

### 4.9 RT‐qPCR Analysis

RT‐qPCR analyses were performed as described before [47]. Sequences of primer pairs provided in Table S3. Data were presented as fold change relative to the control group.

### 4.10 In Vivo Ubiquitination Assay

In vivo ubiquitination assay was conducted using the Ni‐NTA (20561ES08, Yeasen) capture assay as previously described [45].

### 4.11 IHC Analysis

Human gastric cancer tissue microarray (YP‐StcSur2201, Shanghai YBL) containing eighty paired gastric cancer and adjacent tissues was stained with anti‐DTX3L (1:50). Formalin‐fixed paraffin‐embedded sections of the xenografted tumor tissues were prepared and processed as previously described [48], with the primary antibodies being anti‐DTX3L (1:200), anti‐SNAI1 (1:50), anti‐E‐Cadherin (1:100), anti‐Vimentin (1:100) or anti‐Ki67 (1:200). All slides were digitized with Panoramic MIDI scanner (Panoramic MIDI, 3DHISTECH) and analyzed using Image‐Pranti‐o Plus 5.1. H‐scores were calculated by staining intensity.

### 4.12 Flow Cytometry Analysis

For flow cytometry, 1 × 10^6^ cells were first blocked with 5 µl Human TruStain FcX (#422301, Biolegend) for 10 min, then incubated with 5 µl each of FITC‐anti‐CD44 and APC‐anti‐CD133 on ice in the dark for 20 min. After washing, samples were acquired on the BD FACS Calibur flow cytometer and analyzed with FlowJo 10.8.1. For cell sorting, cells were stained as above and isolated into CD44^+^CD133^+^ and CD44^−^CD133^−^ populations using BD FACSAria SORP instrument.

### 4.13 Sphere Formation

Cells were suspended in DMEM/F12 supplemented with 10 ng/ml basic fibroblast growth factor (HY‐P5321, MCE), 20 ng/ml epidermal growth factor (HY‐P7109, MCE), 20 ng/ml leukemia inhibitory factor (HY‐P7049, MCE), 1% B27 (#17504044, Gibco), 5 µg/ml insulin (I3536, Merck), 0.1% Bovine Serum Albumin (B2064, Merck), and 1% penicillin/streptomycin (FG101‐01, Transgen). Cells were seeded at 5000 cells/well in Corning ultra‐low‐attachment 6‐well plate (#150628, Thermo). After two weeks, tumor spheres were imaged and diameters quantified with the MuviCyte cellular imaging system (MuviCyte, PerkinElmer).

### 4.14 Extreme Limiting Dilution Analysis (ELDA)

Cells were seeded at 1, 5, 24, 120, 600, or 3000 cells per well (16 replicates each) in Corning ultra‐low‐attachment 96‐well plates. After two weeks, wells containing spheres were counted and the self‐renewal frequency was calculated with online ELDA tool (http://bioinf.wehi.edu.au/software/elda/) [49].

### 4.15 Bioinformatical Analysis

The clusterProfiler R package was used to run GSEA and compare hallmark pathways between *DTX3L*‐high and *DTX3L*‐low tumor cohorts (with mean cut‐off). Results were visualized with gseaplot2() from enrichplot. For survival analysis, the survival R package was employed, and the optimal DTX3L expression cut‐point was determined with surv_cutpoint(). Kaplan‐Meier survival curves were plotted with survfit().

### 4.16 Dual‐Luciferase Reporter Assay

The *DTX3L* 3‘UTR region spanning nucleotides 1469–1813 containing putative *miR‐135b‐5p* binding sites were inserted into the pmirGLO vector to construct the WT reporter plasmid. Simultaneously, a mutant (MUT) reporter plasmid containing two mutated nucleotides in the putative binding sites was cloned for comparison. HEK293T cells were seeded at a density of 1 × 10^4^ cells/well in 96‐well plate and transfected with 0.1 µg of *DTX3L* 3‘UTR WT or MUT plasmid for 6 h prior to 50 nm *miR‐135b‐5p* mimic or control treatment for 24 h. Cell lysates were prepared, and the dual‐luciferase assay was performed using the Dual‐Luciferase Reporter Assay System (E1910, Promega) according to the manufacturer's protocol. Luminescence was measured using a Multifunction Microplate Reader (Synergy H1, BioTek).

### 4.17 Human Gastric Cancer Organoid Experiment

Human gastric cancer organoids (CCTCC‐GC‐OL001) and complete growth medium (GDM3001) were obtained from the China Center for Type Culture Collection, with ethics approval number B2021‐449R. Organoid culture, maintenance, and lentiviral transduction were performed as previously described [50, 51]. For lentiviral transduction, dissociated organoids were transduced with lentivirus at a multiplicity of infection of 5 in the presence of 1 µg/ml polybrene. The organoids‐lentivirus suspension was centrifuged at 600 × g for 1 h, followed by incubation at 37°C for 4 h. Organoids were then pelleted at 350 × *g* for 3 min, resuspended in Matrix‐Gel (C0396, Beyotime), and plated into 24‐well plates. After 3 days of recovery, organoids were selected with 2 µg/ml puromycin and subsequently cultured in Matrix‐Gel domes overlaid with complete organoid medium for indicated time points. Bright‐field images of organoids were acquired at indicated time points using an inverted microscope (DMi8, Leica). Organoid diameters were measured using Leica Application Suite X (v3.7.4).

### 4.18 Statistical Analysis

Statistical analysis was performed using SPSS (version 31). Results were presented as mean ± SD of at least three independent experiments. Independent‐samples t‐test was employed for comparisons between two groups. For multiple group comparisons, one‐way ANOVA was conducted with Fisher's LSD post‐hoc test. The chi‐square test was used to evaluate differences in the distribution of invasion status in the zebrafish xenograft model. *P* value of less than 0.05 was considered statistically significant. All blots shown were representative of three or more independent experiments.

## Author Contributions

Zhen Wang, Yang Chen, and Zhen Li conceived of and designed the study. Yang Chen, Zhen Li, Jiajia Shen, Jingyu Lin, and Rui Zhang performed experiments and acquired data; Yang Chen performed bioinformatical analyses; Zhen Wang, Yang Chen, Zhen Li, Jiajia Shen, Jingyu Lin, Xiaoli Zhao, and Ying Han analyzed and interpreted the data. Yang Chen, Zhen Li, and Zhen Wang drafted the manuscript. All authors have reviewed and approved the submission.

## Funding

This project is supported by grants from the National Natural Science Foundation of China (82473100) and the CAMS Innovation Fund for Medical Sciences (No. 2025‐2025I2MKJ019 to ZW).

## Conflicts of Interest

The authors declare no conflicts of interest.

## Supporting information




**Supporting File**: advs75262‐sup‐0001‐SuppMat.docx.

## Data Availability

The transcriptomic data used for survival analysis and GSEA in the present study are available through dataset of GEO, TCGA and RNA‐seq of human cancer cell lines [52]. All raw data generated in this study are available upon request from the corresponding author.
