# Supplementary material for: DTX3L Inhibits the EMT, Metastasis, and Stem‐Like Features of Gastric Cancer Through Promoting GSK‐3β Dependent SNAI1 Decay
Source: Adv Sci (Weinh). 2026 Apr 13:e24036. Online ahead of print. doi: 10.1002/advs.202524036 (PMC13334627; doi:10.1002/advs.202524036)
Supplement: Supplementary file 1 — Supporting File: advs75262‐sup‐0001‐SuppMat.docx. [file ADVS-9999-e24036-s001.docx]

**DTX3L inhibits the EMT, metastasis and stem-like features of gastric cancer through promoting GSK-3β dependent SNAI1 decay**

Yang Chen^1#^, Zhen Li^1#^, Jiajia Shen^1^, Jingyu Lin^1^, Xiaoli Zhao^1^, Rui Zhang^1^, Ying Han^1^, Zhen Wang^1^*

1 Department of Biochemistry, Institute of Medicinal Biotechnology, Chinese Academy of Medical Sciences & Peking Union Medical College, Beijing, China.

# These authors contributed equally to this work.

*Correspondence to: Zhen Wang, 1# Tian Tan Xi Li, Beijing 100050, P. R. China.

Tel: 86-10-63165289, Email: [wangzhen@imb.pumc.edu.cn](mailto:wangzhen@imb.pumc.edu.cn)

**Running Title:** DTX3L suppresses EMT and metastasis via degrading SNAI1 **Keywords:** DTX3L, SNAI1, EMT, Ubiquitylation, Gastric Cancer


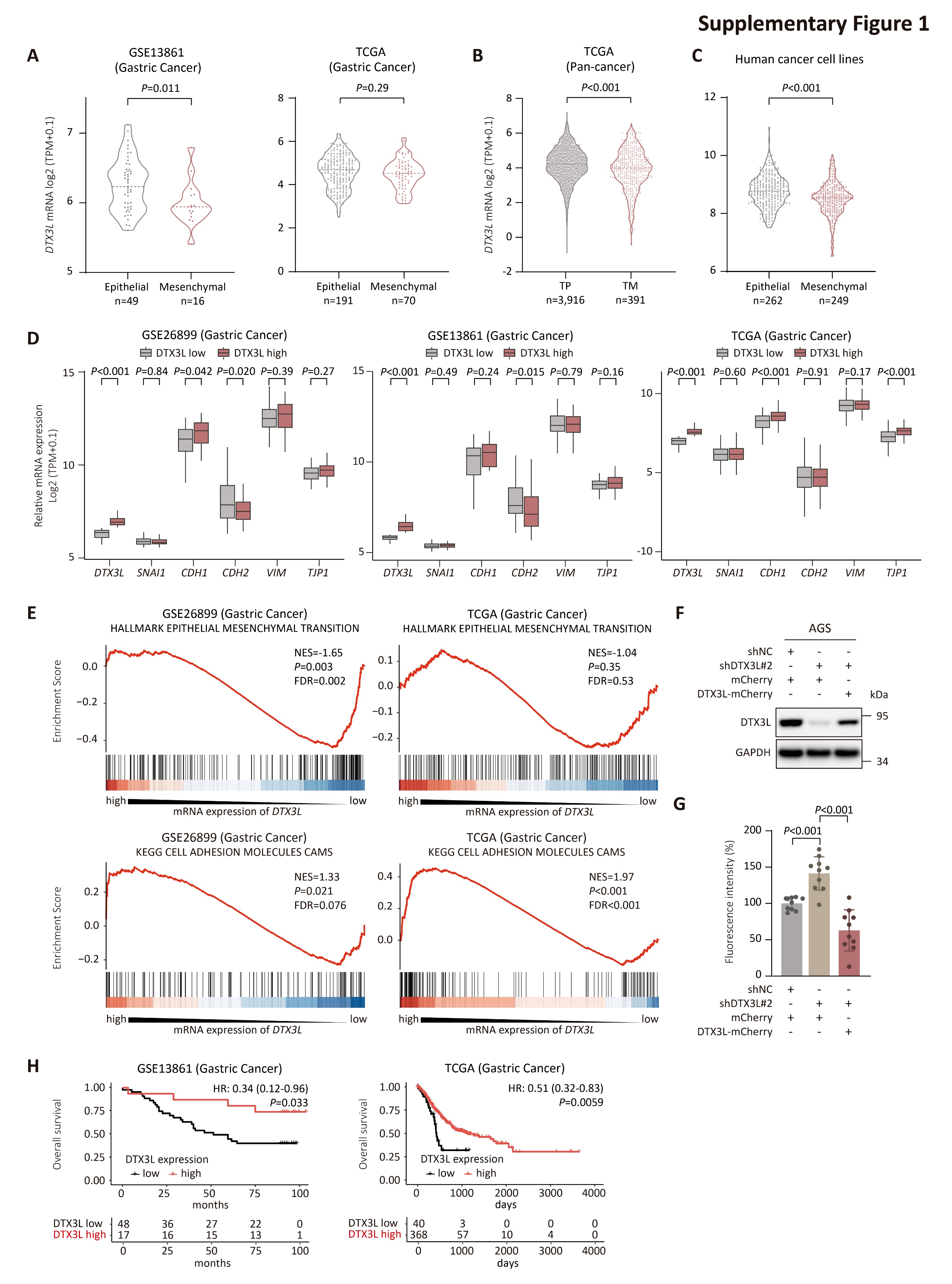


**Supplementary Figure S1.** DTX3L expression is negatively correlated with EMT traits in gastric cancer, with its depletion enhancing metastasis in zebrafish tumor model. (A-C) *DTX3L* mRNA expression levels in different cancer datasets from GSE13861 and TCGA (A), TCGA pan-cancer cohorts (B), and a publicly available RNA-seq dataset of human cancer cell lines in CCLE (C). TP, Primary Solid Tumor. TM, Metastatic. (D) Expression levels of *DTX3L* and EMT markers in the gastric cancer data from GSE26899, GSE13861 and TCGA. (E) GSEA analysis of the gastric cancer data from GSE26899 and TCGA. (F) IB analysis of the mCherry-labeled AGS cells expressing different levels of DTX3L. (G) Fluorescence intensities of AGS cells in zebrafish larva at 5 dpf (n = 10). **(**H) The Kaplan-Meier plots showing the overall survival of the gastric cancer patients correlated with *DTX3L* expression in GSE13861 and TCGA cohorts.


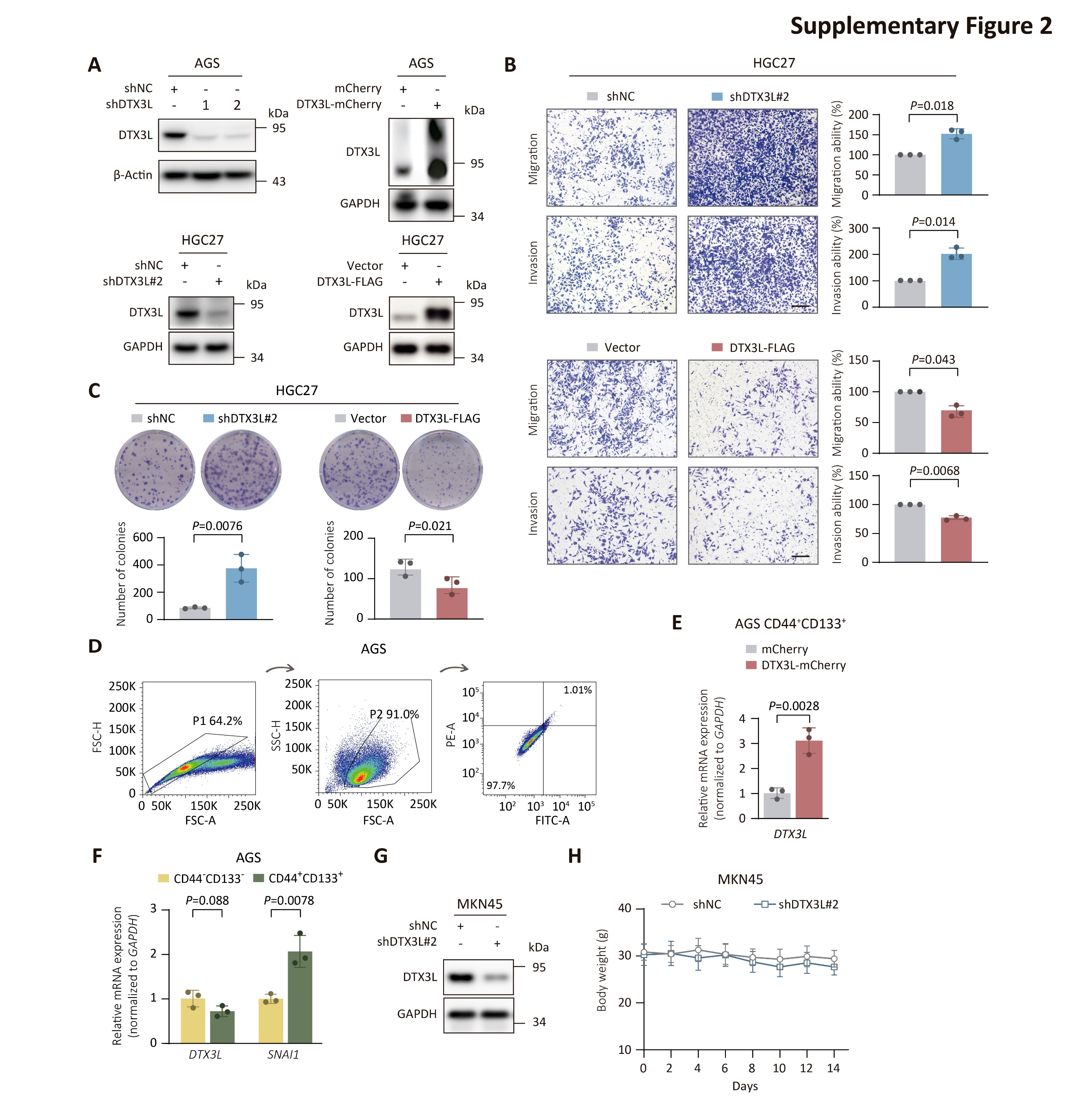


**Supplementary Figure S2.** DTX3L inhibits the migration, invasion, proliferation and stem-like phenotypes of gastric cancer. (A) IB analyses of DTX3L levels in AGS and HGC27 cells upon knockdown or overexpression with indicated vector. (B) Migration and invasion assays for HGC27 cells expressing shDTX3L#2 or DTX3L-FLAG with respective control groups. Scale bar: 200 μm. (C) Colony formation assays for HGC27 cells as in (B). (D) Isolation of CD44⁺CD133⁺ subpopulation AGS cells by flow cytometry. (E) RT-qPCR analysis of the *DTX3L* gene levels of the CD44⁺CD133⁺ AGS cells upon overexpression. **(**F) RT-qPCR analysis of *DTX3L* and *SNAI1* mRNA levels between CD44^+^CD133^+^ and CD44^-^CD133^-^ AGS cell subpopulations. **(**G) IB data of the MKN45 cells used for animal study. (H) The body weights of indicated NSG mice measured every two days during the time (n = 5 per group). Representative images of three independent experiments were shown (B, C).

**
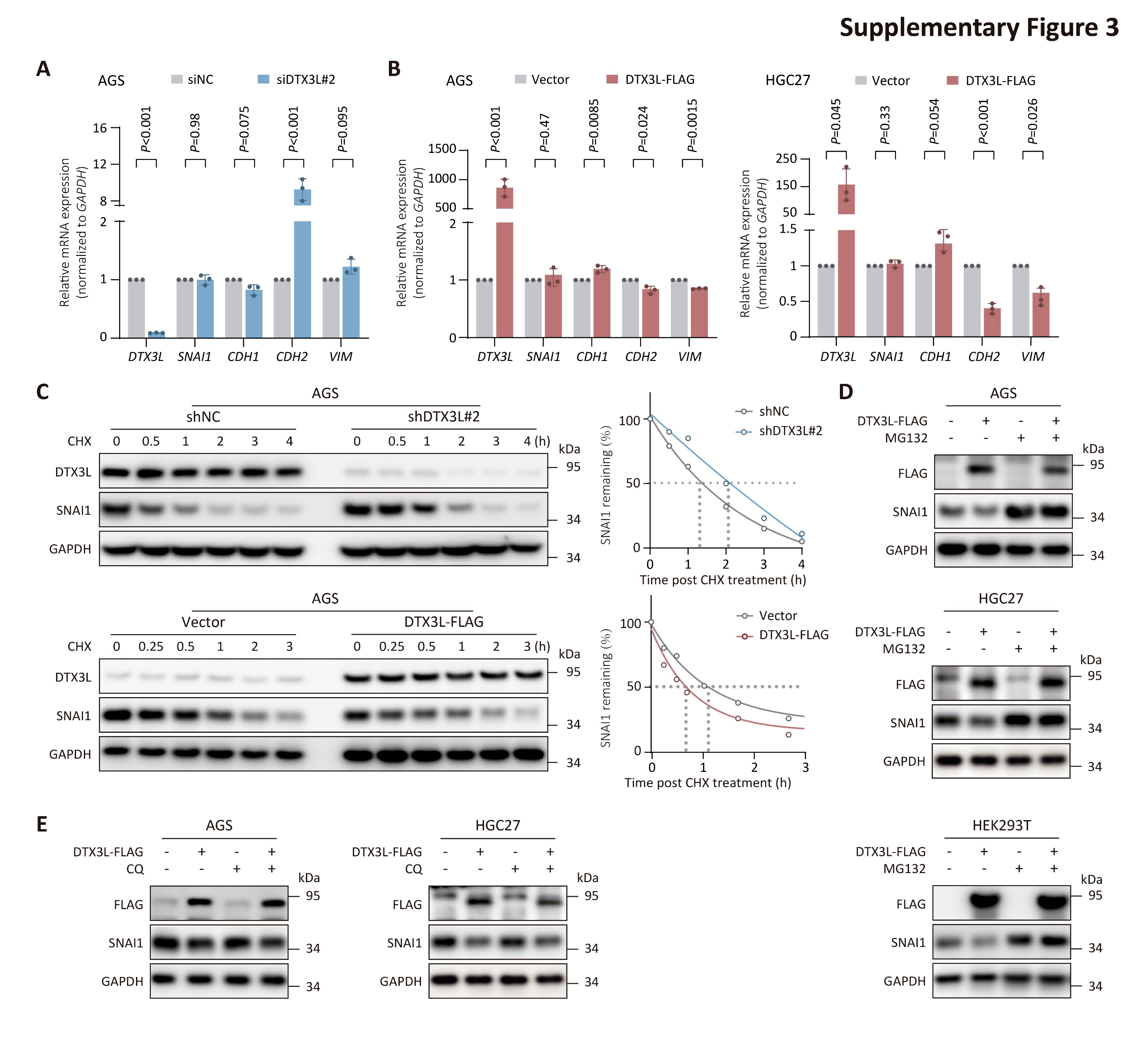
**

**Supplementary Figure S3.** DTX3L inhibits EMT and negatively regulates SNAI1 protein levels via the UPS. (A-B) Cells were transfected with siDTX3L#2 or siNC (A), or DTX3L-FLAG or Vector (B) for 48 h, then subjected to RT-qPCR analyses. (C) AGS cells expressing shDTX3L#2 or shNC, or DTX3L-FLAG or empty vector, were treated with 50 μg/mL CHX for the indicated time interval and subjected to IB analyses. Protein half-life curve was shown in the right panel. (D-E) Cells expressing DTX3L-FLAG or vector for 48 h by transient transfection were treated with 20 μM MG132 (D) or 50 μM CQ (E) for 6 h prior to IB analyses.

**
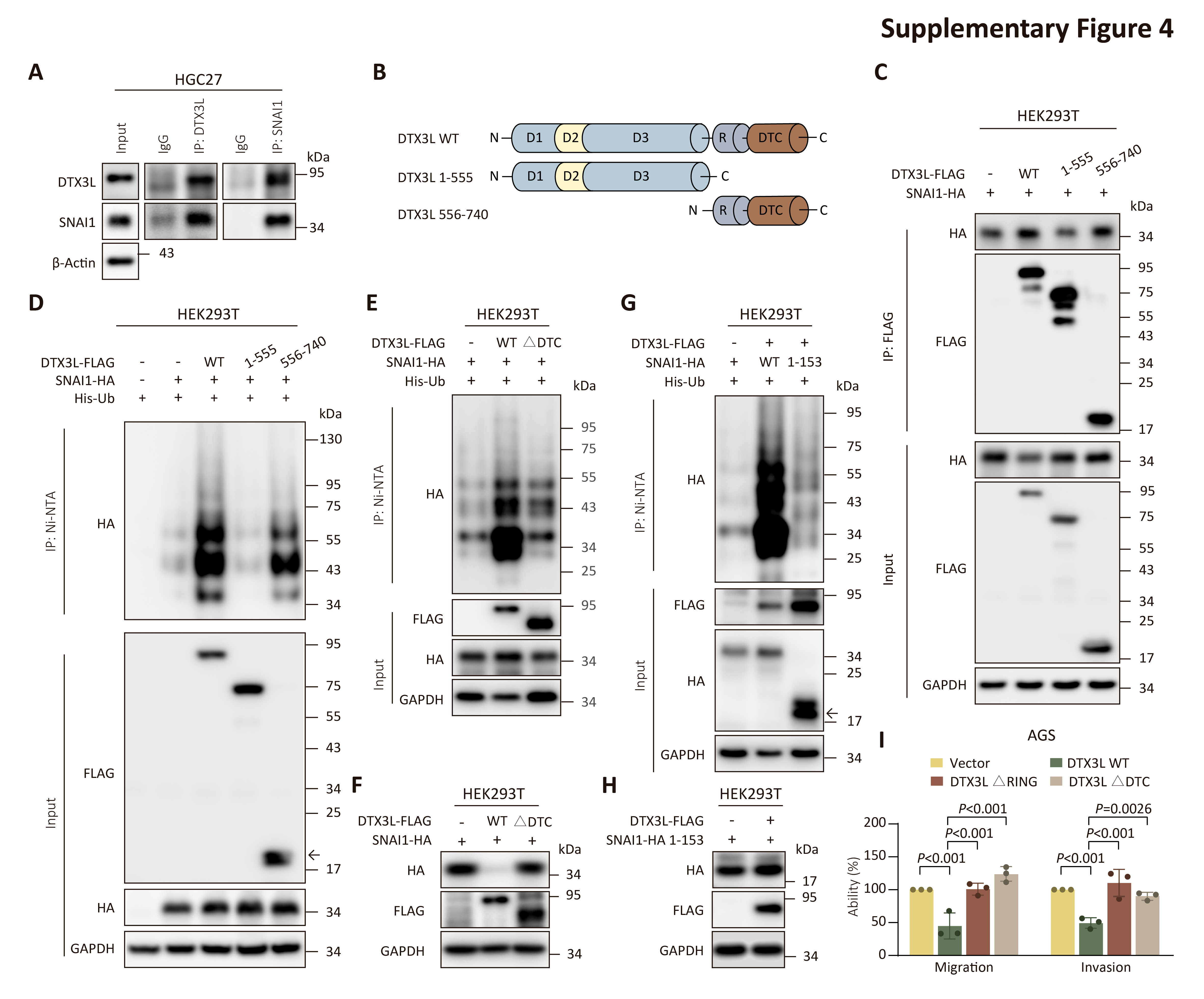
**

**Supplementary Figure S4.** DTX3L interacts with and ubiquitylates SNAI1. (A) HGC27 cells lysates were subjected to IP using anti-DTX3L and anti-SNAI1 antibody or IgG control, followed by IB analyses to detect the interaction between DTX3L and SNAI1. (B) Schematic representation of DTX3L N-and C-terminal deletion mutants. (C) HEK293T cells were transfected with indicated plasmids for 48 h prior to IP and IB analyses. (D) HEK293T cells were co-transfected with indicated plasmids for 48 h. The cells lysates were purified using Ni-NTA beads and analyzed by IB analyses. (E-H) IP-IB (E, G) or IB (F, H) analyses of the HEK293T cells expressing indicated plasmids. (I) Quantification of the transwell assays as in Fig. 4J. Arrow indicates target band.

**
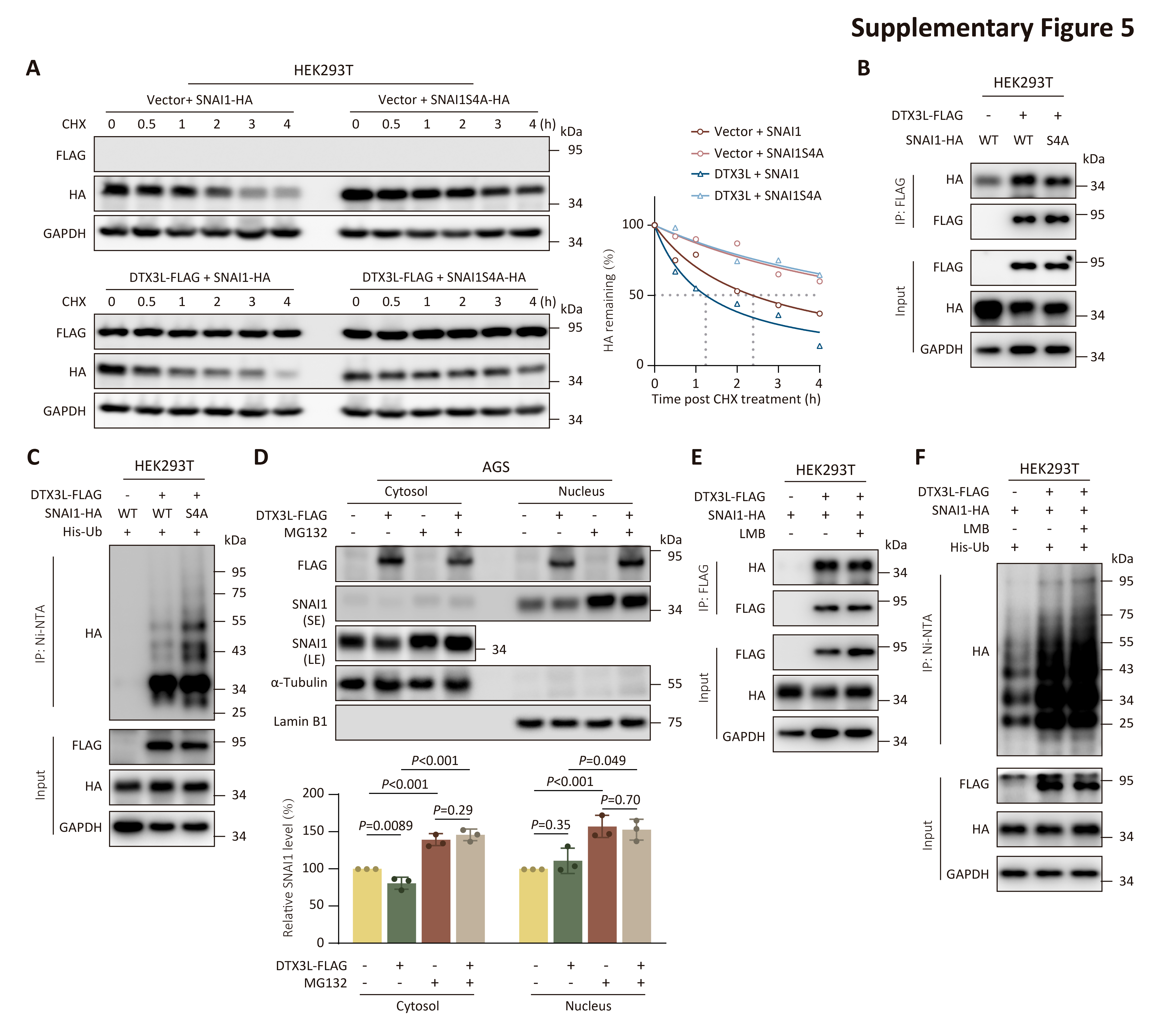
**

**Supplementary Figure S5.** GSK-3β dependent phosphorylation is essential for DTX3L-mediated SNAI1 degradation. (A) HEK293T cells transfected with indicated plasmids for 48 h followed by treatment with 50 μg/mL CHX for indicated time interval before subjected to IB analyses. SNAI1 protein half-lives are shown in the right panel. (B-C**)** Pull-down assays with anti-FLAG (B) or Ni-NTA (C) in HEK293T cells expressing the indicated plasmids. (D**)** AGS cells were transfected with DTX3L-FLAG for 48 h and treated with 20 μM MG132 for 6 h. Nucleic and cytoplasmic fractions were isolated, and then subjected to IB analyses. Protein levels were quantified and plotted in the lower panel. SE: short exposure. LE: long exposure. (E-F) HEK293T cells were transfected with the indicated plasmids for 48 h and then exposed to 100 ng/mL LMB for 2 h, followed by pull-down assays with anti-FLAG (E) or Ni-NTA (F).

**
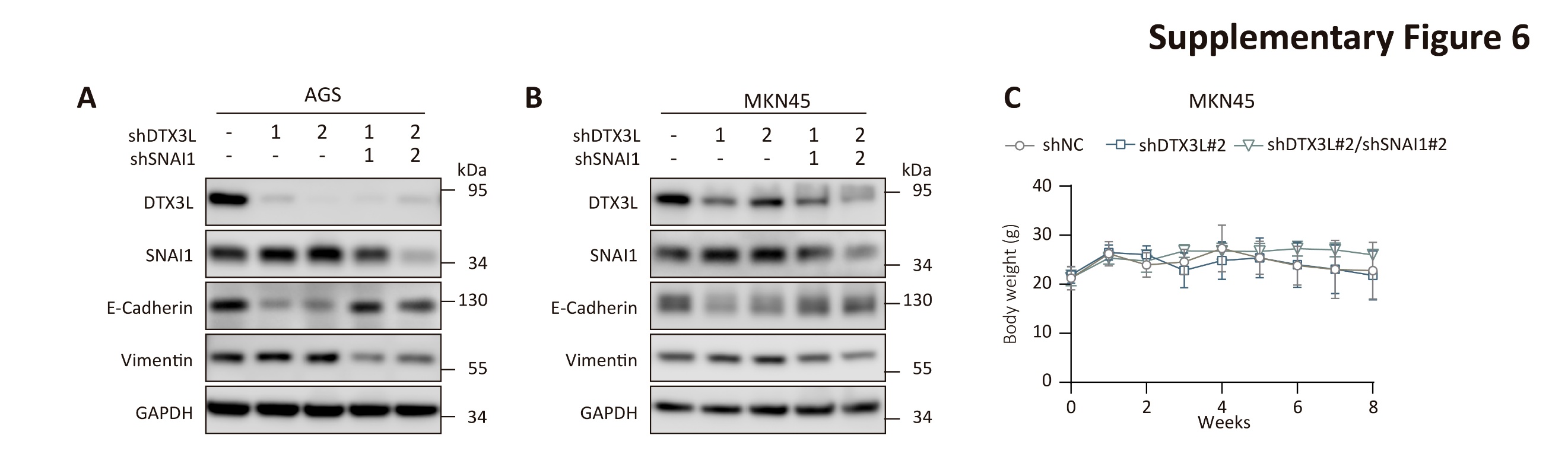
**

**Supplementary Figure S6.** SNAI1 deficiency abrogates the enhanced metastasis of DTX3L-depleted gastric cancer cells. (A-B) IB analyses of the indicated proteins in AGS and MKN45 cells stably expressing indicated shNC, shDTX3L or shDTX3L/shSNAI1. (C) The body weights of BALB/c nude mice as in Fig. 6E were measured every week during the time (n = 5-6).

**
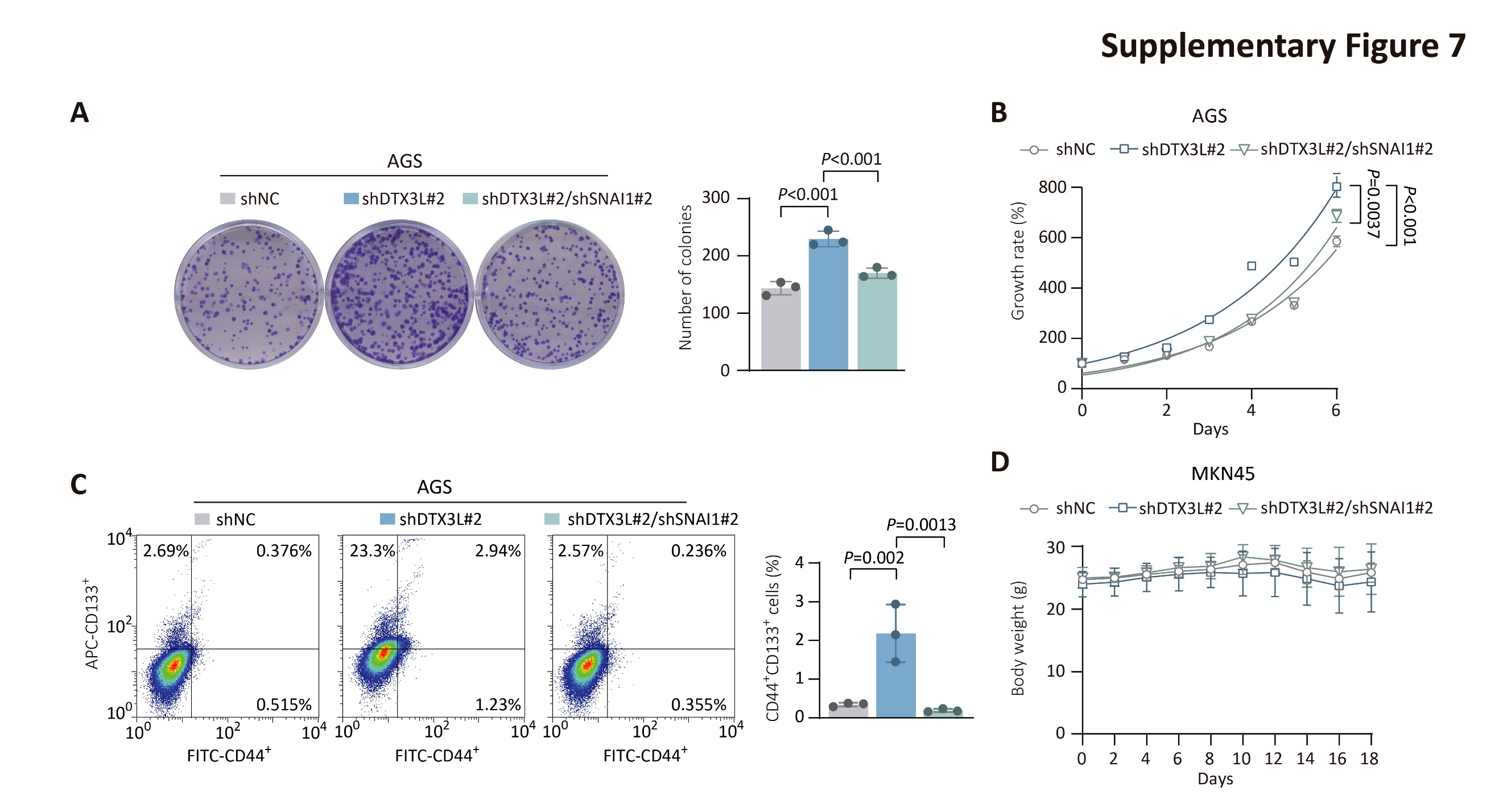
**

**Supplementary Figure S7.** SNAI1 deficiency abrogates the enhanced proliferation of DTX3L-depleted gastric cancer cells. **(**A**)** Colony formation assay of the AGS cells expressing shNC, shDTX3L#2, or shDTX3L#2/shSNAI1#2. Representative images of three independent experiments are shown in the left panel, with quantification demonstrated in the right panel. **(**B**)** Cell proliferation curve of the AGS cells as in (A). (C**)** The proportion of CD44^+^CD133^+^ cells in AGS cells stably expressing shNC, shDTX3L#2 alone or in combination with shSNAI1#2 was analyzed using flow cytometry. (D) The body weights of BALB/c nude mice as in Fig. 7D (n = 6).


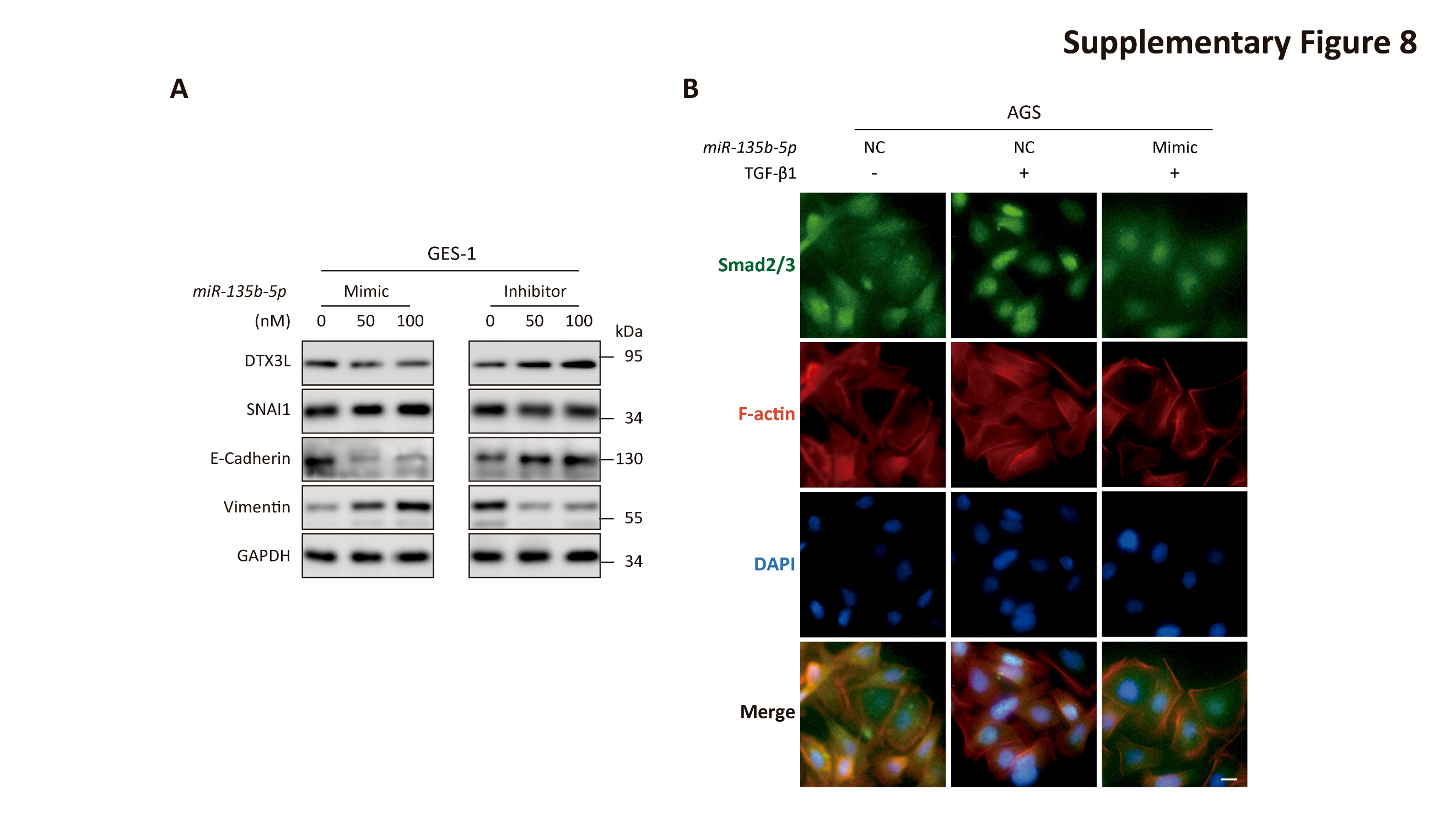


**Supplementary Figure S8. TGF-**β1 **and *miR-135b-5p* exhibit mutual regulatory interactions**. (A) IB of the protein levels in GES-1 cells transfected with *miR-135b-5p* mimic or inhibitor for 48 h. (B) AGS cells were transfected with 50 nM ***miR-135b-5p*** mimic or NC mimic for 24 h, followed by treatment with TGF-β1 (2.5 ng/mL) for 1 h. IF staining was performed to analyze the expression/localization of Smad2/3 (green) and F-actin (red). Scale bar: 10 μm.

**Supplementary Table S1. Information of antibodies**

| **Antigens** | **Company (Catalog#)** | **Host species** | **Application** |
| --- | --- | --- | --- |
| Alexa Fluor® 488-labeled Goat  Anti-Mouse IgG | ZSGB-BIO (ZF-0512) | Goat | IF |
| Alexa Fluor® 594-labeled Goat  Anti-Rabbit IgG | ZSGB-BIO (ZF-0516) | Goat | IF |
| APC anti-human CD133 | Biolegend (372805) | Mouse | FC |
| DTX3L | CST (14795) | Rabbit | IB, IP |
| DTX3L | Abmart (PC14438) | Rabbit | IHC, IF |
| DYKDDDDK (FLAG) | CST (14793) | Rabbit | IB, IP |
| E-Cadherin | CST (3195) | Rabbit | IB, IF, |
| FITC anti-human CD44 | Biolegend (338803) | Mouse | FC |
| FITC-labeled Goat Anti-Rabbit IgG | ZSGB-BIO (ZF-0311) | Goat | IF |
| GAPDH | Proteintech (60004-1-Ig) | Mouse | IB |
| HA | CST (3724) | Rabbit | IB, IP |
| HRP-labeled Goat Anti-Mouse IgG | Jackson Inmuno Research (115-545-003) | Goat | IB |
| HRP-labeled Goat Anti-Rabbit IgG | Jackson Inmuno Research (111-545-003) | Goat | IB |
| Ki-67 | Proteintech (27309-1-AP) | Rabbit | IHC |
| Lamin B1 | Abcam (Ab133741) | Rabbit | IB |
| N-Cadherin | CST (13116) | Rabbit | IB |
| Smad2/3 | CST (8685T) | Rabbit | IF |
| SNAI1 | CST (3879) | Rabbit | IB, IP |
| SNAI1 | Abmart (TA6032) | Rabbit | IHC, IF |
| Vimentin | CST (5741) | Rabbit | IB, IF |
| α-Tubulin | Selleck (F0063) | Rabbit | IB |
| β-Actin | Proteintech (66009-1-Ig) | Mouse | IB |

Abbreviations: IB, immunoblotting; IHC, immunohistochemistry; IF, immunofluorescence; IP, immunoprecipitation; FC, flow cytometry.

**Supplementary Table S2. RNAs and oligonucleotides Sequences**

| **Name** | **Sequence (5'-3')** |
| --- | --- |
| siDTX3L#1 | GCACCATTGTGATTACTTA |
| siDTX3L#2  shDTX3L#1  shDTX3L#2  shSNAI1#1  shSNAI1#2 | GCAGCAAGGTTTCTGAGAA  CGCGTATTAGGAGTCTCAGATTTCAAGAGAATCTGAGACTCCTAATACGCG  CTGCATACTTGCCTGATAATATTCAAGAGATATTATCAGGCAAGTATGCAG  GCGAGCTGCAGGACTCTAATCTTCAAGAGAGATTAGAGTCCTGCAGCTCGC  ACTCAGATGTCAAGAAGTACCTTCAAGAGAGGTACTTCTTGACATCTGAGT |
| *miR-135b-5p* mimic | Sense: UAUGGCUUUUCAUUCCUAUGUGA  Antisense: ACAUAGGAAUGAAAAGCCAUAUU |
| *miR-135b-5p* inhibitor | UCACAUAGGAAUGAAAAGCCAUA |

**Supplementary Table S3. Primers sequences of genes**

| **Gene** | **Forward sequence (5’-3’)** | **Reverse sequence (5’-3’)** | **Usage** |
| --- | --- | --- | --- |
| *CD133* | AGTCGGAAACTGGCAGATAGC | GGTAGTGTTGTACTGGGCCAAT | qPCR |
| *CD24* | CTCCTACCCACGCAGATTTATTC | AGAGTGAGACCACGAAGAGAC | qPCR |
| *CD44* | CTGCCGCTTTGCAGGTGTA | CATTGTGGGCAAGGTGCTATT | qPCR |
| *CD90* | ATCGCTCTCCTGCTAACAGTC | CTCGTACTGGATGGGTGAACT | qPCR |
| *CDH1* | ATTTTTCCCTCGACACCCGAT | TCCCAGGCGTAGACCAAGA | qPCR |
| *CDH2* | TGCGGTACAGTGTAACTGGG | GAAACCGGGCTATCTGCTCG | qPCR |
| *DTX3L* | CCAGGTTATGAGTCCTTTGGCAC | TGCAGTTCGCTGTATTCCAGGG | qPCR |
| *GAPDH* | CATGAGAAGTATGACAACAGCCT | AGTCCTTCCACGATACCAAAGT | qPCR |
| *miR-135b-5p* | GTCGTATCCAGTGCAGGGTCCGAGGTATTCGCACTGGATACGACTCACAT | | RT-PCR |
| *miR-135b-5p* | TATGGTATGGCTTTTCATTCCT | GTGCAGGGTCCGAGGTATT | qPCR |
| *NANOG* | TTTGTGGGCCTGAAGAAAACT | AGGGCTGTCCTGAATAAGCAG | qPCR |
| *OCT4* | GGGAGATTGATAACTGGTGTGTT | GTGTATATCCCAGGGTGATCCTC | qPCR |
| *SNAI1* | ACTGCAACAAGGAATACCTCAG | GCACTGGTACTTCTTGACATCTG | qPCR |
| *SOX2* | GCCGAGTGGAAACTTTTGTCG | GGCAGCGTGTACTTATCCTTCT | qPCR |
| U6 | AACGCTTCACGAATTTGCGT |  | RT-PCR |
| *U6* | GCTTCGGCAGCACA | AACGCTTCACGAATTTGCGT | qPCR |
| *VIM* | TGCCGTTGAAGCTGCTAACTA | CCAGAGGGAGTGAATCCAGATTA | qPCR |
